# ClinIAN: Clinically Informed Attention Network

**DOI:** 10.1093/bioadv/vbag254

**Published:** 2026-09-09

**Authors:** Florian König, Jonas C Ditz, Elham Shamsara, Pontus Hedberg, Iuri Fanti, Pontus Nauclér, Luca Carioti, Andreas Walker, Milosz Parczewski, Francis Drobniewski, Francesca Ceccherini-Silberstein, Maurizio Zazzi, Björn-Erik Ole Jensen, Anders Sönnerborg, Nico Pfeifer

**Affiliations:** Institute for Bioinformatics and Medical Informatics (IBMI), University of Tübingen, Tübingen 72076, Germany; Institute for Bioinformatics and Medical Informatics (IBMI), University of Tübingen, Tübingen 72076, Germany; Institute for Bioinformatics and Medical Informatics (IBMI), University of Tübingen, Tübingen 72076, Germany; Division of Infectious Diseases, Department of Medicine Huddinge, Karolinska Institutet, Stockholm 17177, Sweden; EuResist Network GEIE, Roma 00152, Italy; Department of Infectious Diseases, Karolinska University Hospital, Stockholm 17177, Sweden; Division of Infectious Diseases, Department of Medicine, Solna, Karolinska Institutet, Stockholm 17177, Sweden; Department of Experimental Medicine, University of Rome Tor Vergata, Rome 00133, Italy; Institute of Virology, Medical Faculty and University Hospital Düsseldorf, Heinrich Heine University Düsseldorf, Düsseldorf 40225, Germany; Department of Tropical Infectious Diseases and Immune Deficiency, Pomeranian Medical University in Szczecin, Szczecin 70-204, Poland; Department of Infectious Disease, Imperial College, London, W12 0NN, United Kingdom; University Hospital Dorset, Poole Hospital, Dorset, BH15 2JB, United Kingdom; Department of Experimental Medicine, University of Rome Tor Vergata, Rome 00133, Italy; Department of Medical Biotechnologies, University of Siena, Siena 53100, Italy; Department of Gastroenterology, Hepatology and Infectious Diseases, Medical Faculty and University Hospital Düsseldorf, Heinrich Heine University, Düsseldorf 40225, Germany; Division of Infectious Diseases, Department of Medicine Huddinge, Karolinska Institutet, Stockholm 17177, Sweden; Department of Clinical Microbiology, Karolinska University Hospital, Stockholm 141 86, Sweden; Institute for Bioinformatics and Medical Informatics (IBMI), University of Tübingen, Tübingen 72076, Germany

## Abstract

**Motivation:**

Artificial Neural Networks (ANNs) hold promise in predicting disease severity from viral protein sequences. To gain clinical insights, the models must adhere to two key factors: first, they need to be interpretable, as using black-box models within a healthcare setting poses risks, and second, they should integrate viral and clinical features to correct for biases within the data. We propose ClinIAN (Clinically Informed Attention Network), an inherently interpretable end-to-end learning model that effectively combines clinical and sequence parameters. We evaluate ClinIAN within the context of the challenging task of predicting the severity of SARS-CoV-2 infection, but it could be applied to other medical and biological contexts, with sequence and tabular features, such as bacterial infections or cancer research.

**Results:**

We evaluate the performance capabilities of ClinIAN in predicting SARS-CoV-2 severity using a subset of the EuCARE hospitalized cohort. We show ClinIAN’s ability to capture biologically relevant features across multiple layers of resolution while retaining stable state-of-the-art performance.

**Availability and implementation:**

The code is available on Zenodo and on GitHub.

## 1 Introduction

The impact of SARS-CoV-2 and the viral infection COVID-19 it causes continues to affect healthcare systems worldwide, even after more than five years since it was first identified in December 2019. As of 10 November 2024, the World Health Organization (WHO) reported more than 776.8 million confirmed cases and more than 7 million deaths attributed to the disease (WHO 2024). Although the pandemic has transitioned to an endemic phase, this situation presents a valuable opportunity to use data to improve preparedness for future health emergencies and mitigate medical and socio-economic challenges (Seabra *et al.* 2024). Several projects and initiatives have gathered and analyzed data about the pandemic. Among these efforts is the European Cohorts of Patients and Schools to Advance Response to Epidemics (EuCARE) (https://eucareresearch.eu) project, which has amassed substantial data to support pandemic response while also examining its effects on educational institutions and post-acute sequelae of SARS-CoV-2 (Nicola *et al.* 2020, Bai *et al.* 2024, Bellerba *et al.* 2024).

These extensive data repositories can help understand the mechanisms of viral infections, which is essential for effective protection, particularly in identifying markers that predict disease severity. Various factors, including host characteristics (age, sex, comorbidities, lifestyle, genetic predispositions) (Hedberg *et al.* 2024) and pathogenic elements (viral variant virulence) (Abdullah *et al.* 2022), as well as interventions (Mohammed *et al.* 2022) and behavioral influences, must be considered. However, assessing all of these influencing factors in a laboratory setting can be very expensive and often unfeasible.

Computational methods can support these efforts. In this space, artificial neural networks (ANN) have become crucial tools across a variety of applications due to their ability to identify nuanced relationships in large and complex datasets. ANNs can perform end-to-end learning for feature extraction and prediction, reducing the need for labor-intensive data preprocessing. They have also become a staple in biological and medical settings. From image classification and segmentation in biological imaging (Sarvamangala and Kulkarni 2022), protein structure prediction (Jumper *et al.* 2021), epidemiology (Xiang *et al.* 2021), to personalized medicine (MacEachern and Forkert 2021).

Nonetheless, the application of ANNs to medicine requires a clear understanding of the inherent decision-making processes known as interpretable AI. This requirement poses a significant challenge, as ANNs are typically considered black-box models that exhibit strong predictive performances but lack transparency in their decision-making processes.

To address this challenge, several methods have been proposed to understand the processes without altering the model structure, including the so-called *post-hoc* interpretation methods SHAP (Lundberg and Lee 2017) and DeepLIFT (Shrikumar *et al.* 2017). These methods are advantageous because they can be applied without modifications to the training regimen, allowing them to be used with any trained predictor. However, these methods have shown inconsistencies across applications, leading to uncertainty about the validity of the interpretations (Rudin 2019, Bordt *et al.* 2022). Several classical machine learning models, such as linear regression and decision tree methods, are inherently interpretable without the need for post-hoc methods. This *inherent interpretability* has also been successfully applied to ANNs, often leveraging attention mechanisms to enable model transparency (Balcı *et al.* 2023, Ditz *et al.* 2023a, 2023b).

Within biological and medical applications, several approaches have been proposed to predict sequence-to-function relationships from sequencing data, including those available for SARS-CoV-2 (Dey *et al.* 2020). However, existing strategies often focus solely on the spike protein (S), neglecting the influence of other viral proteins. While the spike protein plays a key role in virus-host interactions, other proteins contribute significantly to viral infection dynamics and virulence (Kehrer *et al.* 2023, Walia *et al.* 2024). Additionally, many approaches oversimplify disease progression by focusing solely on viral sequences, thereby overlooking the impact of clinical and epidemiological factors. By excluding these critical features, there is a risk of introducing biases that skew the model towards mutations that could be better understood through clinical data, such as those that do not have a strong effect on mortality but are simply present in a successful variant. Integrating clinical and sequential features promises to enhance insights into the decision-making processes of the model. However, clinical data often present challenges due to its inherent heterogeneity and tabular structure. While ANNs excel in contexts with homogeneous data, they tend to struggle with heterogeneous tabular data. Conversely, decision tree methods, such as Random Forests and XGBoost, have been staples in tabular data analysis, but lack the capacity to learn embeddings within a greater modeling context. Recent advancements have led to ANN approaches capable of effectively handling heterogeneous tabular data while performing on par with or outperforming tree-based approaches (McElfresh *et al.* 2023, Borisov *et al.* 2024). A notable approach is TabNet (Arik and Pfister 2021), a state-of-the-art architecture that generates interpretation masks and feature embeddings, allowing insights into the relative importance of individual clinical attributes.

In this work, we introduce ClinIAN (Clinically Informed Attention Network), a novel solution that effectively integrates clinical tabular and viral amino acid (AA) sequence data using artificial neural networks (ANNs) within an inherently interpretable, end-to-end learnable framework. We design ClinIAN to predict disease severity, leveraging both data modalities. ClinIAN applies TabNet to generate interpretation masks for clinical features and embeddings in later network layers. We incorporate AA sequence data by applying multiple-instance learning (MIL) based attention (Ilse *et al.* 2018) to enable interpretability both within and across proteins.

We benchmark ClinIAN against established baseline methods using data from the hospitalized cohort of the EuCARE project. The EuCARE hospitalized study (Hedberg *et al.* 2023) provides a rich and nuanced dataset that includes both clinical and viral features for a subset of patients, offering an excellent platform for assessing the models’ capabilities. Our analysis reveals the performance and interpretability of ClinIAN across multiple layers of resolution, including patient-level predictors, protein-level contributions, and the influence of specific mutational positions on the predictions. By adopting a multimodal approach to predict medical severity in an interpretable manner, our framework promises to enhance decision-making processes in clinical settings.

## 2 Material and methods

### 2.1 EuCARE whole genome sequencing cohort

We assessed the integration of clinical and viral features within a unified end-to-end trainable model utilizing data from the EuCARE hospitalized study (Hedberg *et al.* 2023). Our analysis is based on data collected as of 9 July 2024. The dataset from the hospitalized study encompasses a broad range of information, including laboratory parameters, patient comorbidities, and clinical patient metadata. For our analysis, we streamlined the parameter set to focus on 15 key factors: age, sex, infecting variant (variant of concern, VOC), year of hospitalization, and comorbidities (cancer, cardiac and cerebrovascular diseases, chronic kidney diseases, chronic liver diseases, chronic lung diseases, diabetes, hypertension, immunosuppression, neurologic diseases including dementia, obesity, and count of vaccinations). We made this decision due to missing data points in other features, ensuring we focus only on factors available to all patients. The primary outcome of interest is defined as *mortality* (within 30-days after infection). In the following, we refer to this variable selection as *metadata*. Further details regarding the distribution of features, labels, and case counts are provided in the supplementary material (Figs SF1 and SF2, available at supplementary material  *Bioinformatics Advances* online).

### 2.2 Data preparation

The subset within the hospitalized cohort that comprises both viral and clinical features, as described above, comprises 6982 samples. The viral sequences are represented as ribonucleic acid (RNA) sequences. To reduce dimensionality in the input space, we translated the sequences into amino acid (AA) sequences, focusing on identifying mutation positions within each viral protein. We applied Nextclade (Aksamentov *et al.* 2021) to align the sequences with the reference Wuhan sequence (Wuhan-Hu-1; Accession: MN908947.3) and identify amino acid mutations within each protein. Subsequently, we refined the set of mutations, retaining only those that occur in at least 0.5% of sequences, thereby reducing potential noise within the data (Table 1).

**Table 1:** This table displays the different mutation counts in the AA sequences of annotated and unannotated open reading frames (ORF) after applying quality control measures.

Proteins	Length	Ins.	Del.	Sub.	#positions
**S**	1273	3	11	69	76
**ORF1a**	4401	0	14	53	66
**ORF1b**	2695	0	0	33	33
**N**	419	0	4	24	25
**ORF9b**	97	0	4	9	12
**ORF3a**	275	0	0	12	11
**ORF8**	121	0	3	3	5
**ORF7a**	121	0	0	5	5
**M**	222	0	0	5	4
**ORF7b**	43	0	0	2	2
**ORF6**	61	0	0	2	2
**E**	75	0	0	1	1

Note that the sum of individual mutations might differ for each #position, as some sequence positions can exhibit multiple mutations (e.g. position 3 in M: D3G and D3N). S, spike protein; E, envelope protein; M, membrane protein; N, nucleocapsid protein.

Ins., Insertion.

Del., Deletion.

Sub., Substitution.

We applied quality control measures to reduce the dataset and retain only high-quality samples. These measures included the removal of samples with a high percentage of ambiguous residues (“X”) in at least one protein (>10%). We also excluded samples that were substantially shorter than the reference sequence (<80%). Sequences containing mutations that led to a stop codon were also discarded. Acknowledging that multiple samples from individual patients may exist over time, we opted to exclude all subsequent sequences for this analysis, as our aim was not to model temporal variations, but rather to investigate the factors contributing to severe hospitalization in the first place. Additionally, we ensured that all clinical features were complete. Detailed steps of the preprocessing are outlined in the supplementary materials (Fig. SF10, available at supplementary material  *Bioinformatics Advances* online). Following these procedures, the final dataset comprised 3328 samples, including 2999 samples from patients categorized as “survival” and 329 samples from patients labeled as “mortality.” We refer to this AA mutation set as *mutations* and to the entire dataset with metadata as the EuCARE whole-genome sequencing (WGS) cohort.

To encode the identified mutations, we employed one-hot encoding. However, it is important to note that the ClinIAN model can be adapted to incorporate positional encodings derived from protein language models (pLMs), as demonstrated in our evaluations. To maintain dimensionality, we substituted positions with the corresponding amino acids from the reference sequences, unless a mutation was present. This encoding for a given protein *p* is denoted as $\eta_{p}$. We adjusted for insertions by expanding the mutation set to include a vector of zeros at the positions of the reference sequence and in all sequences lacking a specific insertion. For the analysis using pLM embeddings, we replace these positional one-hot-encoded vectors with vectors from the pLM. We create positional embeddings for the entire sequences and select the embeddings at the specific positions used in our analysis as input vectors for the model.

While features such as sex and comorbidities are provided categorically, we adhered to the encoding framework proposed by Hedberg *et al.* (2024) for age and vaccinations. In particular, age was stratified into 3 distinct categories (18–49, 50–69, 70+), and vaccination status was classified into four categories (0, 1, 2, and 3 or more doses). The infecting variant was determined using Nextclade (Aksamentov *et al.* 2021), with the assigned Pango lineage (Rambaut *et al.* 2020, O’Toole *et al.* 2021) for each sample mapped to the closest variant within a predefined set that includes the Wuhan strain, as well as the Alpha, Beta, Delta, Gamma, and Omicron variants (BA.1, BA.2, BA.4, BA.5, BA.5.3.1, BN.1, BQ.1.1). For the baseline models, we employed one-hot encoding similarly to the sequence data. Our proposed model, ClinIAN, was designed to effectively handle categorical data without prior encoding by leveraging the capabilities of TabNet.

### 2.3 ClinIAN—Clinically Informed Attention Network

In the following section, we introduce the modeling background of the ClinIAN network structure. We build on two ideas: learning from tabular data for clinical features and using MIL for the mutational features.

#### 2.3.1 ClinIAN_seq_—utilizing mutation data

**Figure 1:**
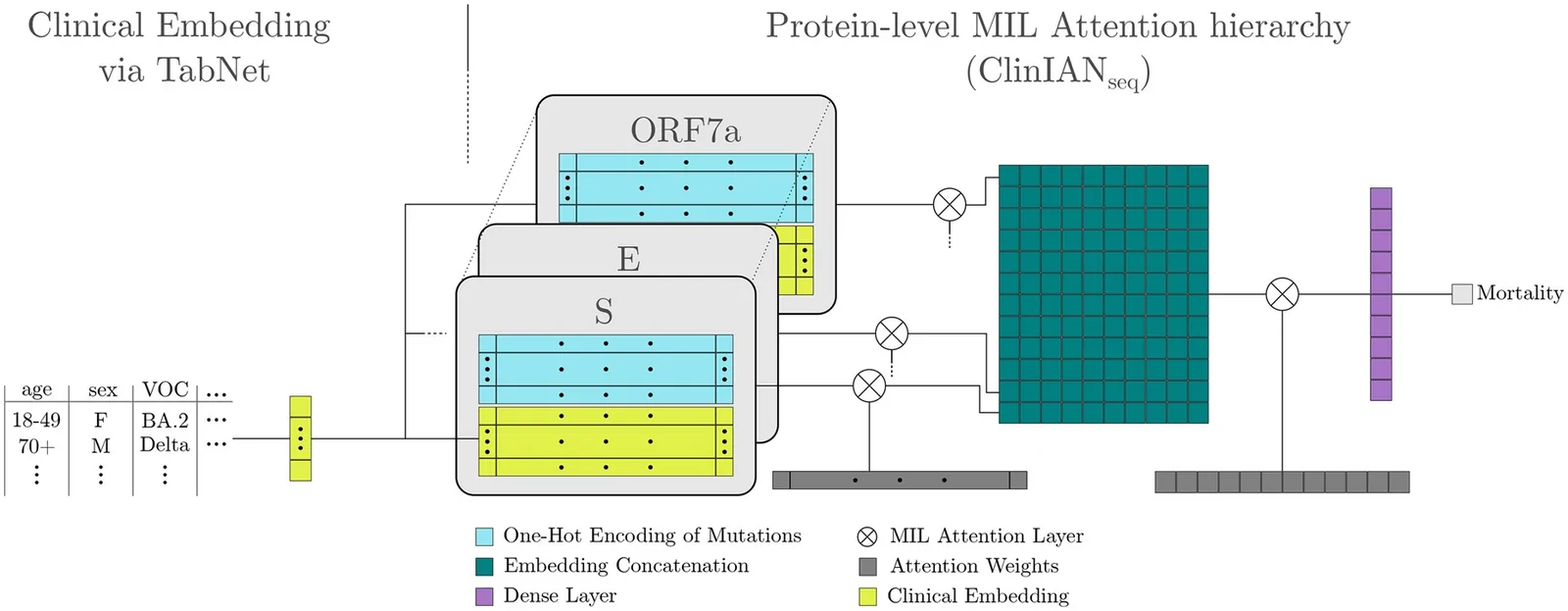
Visualization of the proposed ClinIAN model. ClinIAN consists of a TabNet that embeds clinical features and provides interpretations, as well as a multilevel mutation-based attention model (ClinIAN_seq_). We concatenate the TabNet embedding vector for each mutation position, enabling the model to consider this information for each position. For the proposed purely sequence based model ClinIAN_seq_, we create an embedding matrix for each protein by encoding every mutation position as a one-hot encoded vector. These embeddings are forwarded through a MIL attention layer to produce local attention weights for each mutation in each protein. The second MIL attention layer produces interpretations of the embeddings from the previous attention layers, indicating which protein is most relevant to the final severity prediction.

The first step towards ClinIAN was to develop an inherently interpretable, attention-based model that uses sequence information to predict COVID-19 disease severity. The model consists of two layers, providing different levels of information about its prediction decisions. The right part of Fig. 1 shows a schematic visualization of the model structure. A complete schematic is provided in the supplements (Fig. SF6). Similar hierarchical structures have been introduced before for other tasks (Do and Lähdesmäki 2025).

Our model provides full inherent interpretability by applying attention-based MIL with a gating mechanism (Ilse *et al.* 2018). In general, MIL treats each sample as a bag of instances, with a single label for the bag (Dietterich *et al.* 1997). In MIL, two approaches exist: instance-level MIL and bag-level MIL, with instance-level MIL providing interpretability within models and bag-level MIL achieving stronger performance (Carbonneau *et al.* 2018, Wang *et al.* 2018). The extension of MIL with attention mechanisms has enabled interpretability comparable to that of instance-level MIL while retaining the performance of bag-level MIL (Ilse *et al.* 2018). Within our model, we applied MIL on two separate layers. First, we considered each mutation position as an instance. Therefore, there is a bag for each protein. Each MIL layer provides an embedding, which we then use in a second bag, where each protein embedding is considered as an instance.

The first level of the model applies attention-based MIL for each protein individually. We applied MIL layers for each protein as follows. We considered each protein *p* and its embedding as a bag of instances $H_{p}=\{\eta_{p,1},\ldots ,\eta_{p,N_{p}}\}$ where $N_{p}$ is the number of mutation positions within *p*. We computed the calculated embedding of the bag


(1) $$z_{p}=\sum_{n=1}^{N_{p}}a_{p,n}\eta_{p,n}$$

with


(2) $$a_{p,n}=\frac{\;\text{exp}\;\{w^{T}(\text{tanh}(V\eta_{p,n}^{T})\}}{\sum_{j=1}^{N_{p}}\;\text{exp}\;\{w^{T}(\text{tanh}(V\eta_{p,j}^{T})\}}$$

It was shown that adding a gating approach increases the expressiveness of the layer (Ilse *et al.* 2018). We therefore adapted the calculation of $a_{p,n}$ as follows:


(3) $$a_{p,n}=\frac{\;\text{exp}\;\{w^{T}(\text{tanh}(V\eta_{p,n}^{T})⊙\mathrm{sigm}(U\eta_{p,n}^{T}))\}}{\sum_{j=1}^{N_{p}}\;\text{exp}\;\{w^{T}(\text{tanh}(V\eta_{p,j}^{T})⊙\mathrm{sigm}(U\eta_{p,j}^{T}))\}}$$

The variables $w\in R^{L\times 1}$, $V\in R^{L\times M}$, and $U\in R^{L\times M}$ are parameters of the attention layer and sigm(·) is a sigmoid non-linearity. Applying the MIL attention in this way enables the observation of the feature importance of each mutation within the sequence for the model’s final prediction. The learned embeddings $z_{p}$ are then concatenated to form a new bag of instances *Z*.

The second level of the model utilizes the newly created embedding matrix *Z* as a new bag of instances and applies an attention-based MIL layer again. The embedding for the bag $Z=\{z_{S},\ldots ,z_{\mathrm{ORF}7a}\}$ is calculated in a manner analogous to the prior layer (see Supplement, EQ. SE1).

This enables the feature importance of each complete protein in the prediction task to be evaluated. Our model, therefore, provides multiple layers of resolution within our interpretability scheme to uncover the important features. In our training, we optimized the embedding vector dimension. The activation of the last layer is passed through a linear layer, which classifies the severity of COVID-19 disease.

#### 2.3.2 ClinIAN—incorporating clinical information

We extended the proposed model structure by integrating TabNet (Arik and Pfister 2021) as an upstream module, then combining its outputs within our architecture. TabNet can make predictions using heterogeneous tabular data while providing interpretations of the feature importances. The network consists of multiple sequential attention steps that combine feature selection and prediction, mimicking the approach of tree-based methods. Each step uses a learned attention mask that emphasizes the most salient features for the prediction task. By aggregating these masks across steps, we derive a comprehensive feature-importance map for the clinical metadata. Additionally, we use TabNet to learn an embedding of the clinical metadata within the greater context of our model. The dimensionality of this embedding vector is systematically optimized within our parameter grid.

We concatenated this embedding vector with the mutation at each position across all proteins, thereby enriching the attention-based MIL layers with both sequence and clinical information. This strategic integration served multiple purposes. By appending the embedding vector to each position, we utilize this clinical information to mitigate biased attention toward mutation positions that may be more easily explained by clinical parameters. This adjustment applies to the calculation of attention weights, which are computed instance-wise, thereby ensuring that no inherent bias is introduced toward specific mutations due to variations in clinical vectors. This approach mimics the correction for population structure in linear mixed models (Hoffman 2013, Hake *et al.* 2023). Alternatively, one might consider implementing an additional layer to project the TabNet embeddings onto protein-specific embeddings. However, this approach risks introducing inconsistencies among proteins, attributable to the differing lengths of each protein’s embedding (for instance, the envelope protein (E) has an embedding dimension of one, while the spike protein (S) has an embedding dimension of 76, as detailed in Table 1).

Subsequently, these concatenated features are processed through an analogous model architecture to that utilized in the sequence-based approach. Figure 1 displays the full ClinIAN model structure. The entire model is designed for end-to-end training, providing interpretability for clinical features through TabNets’ structure and the viral sequence interpretations described above. We optimized ClinIAN using the additional sparsity loss term suggested for TabNet (Arik and Pfister 2021) with $L=L_{\text{prediction}}-\lambda L_{\text{sparsity}}$ where λ controls the strength of the sparsity regularization (see supplement EQ. SE3).

This strategic integration of tabular confounders into a hierarchical model differentiates ClinIAN from other multimodal approaches that first encode modalities independently, such as MOMA (Moon and Lee 2022). The structure allows for a principled approach: first analyzing the influence of whole proteins, then enabling deeper analysis of the correlation between mutations and the outcome.

## 3 Results

We trained the models using the EuCARE WGS cohort, which includes viral sequences, to predict COVID-19 mortality. Our approach’s performance was compared against established baseline methods, specifically Support Vector Machine (SVM) and Random Forests. We further incorporated the state-of-the-art method MOMA (Moon and Lee 2022) in our comparison. To ensure a fair evaluation in performance reporting, we used a 10x10 Nested Cross-Validation (CV) strategy in our training pipeline, optimizing hyperparameters for both the baseline and proposed models via grid search. Detailed information about the grid parameters can be found in the supplementary materials (Sec. S3, available at supplementary material  *Bioinformatics Advances* online). To maintain consistency across the observed data at each iteration, we provided an index file containing the indices for the inner and outer folds. This ensured that all models were trained on the same data during each cross-validation fold, and the same test sets were used for evaluation. Additionally, we corrected for class imbalances within training folds with our evaluated parameter grid either with random over-sampling or by applying the class-balance loss (Cui *et al.* 2019) for the PyTorch models and with class-weights for the sklearn models. The comparison method MOMA was trained analogously with the same training loop and optimized according to the authors suggestions (see Sec. S3, available at supplementary material  *Bioinformatics Advances* online). We evaluated model performance with the Matthews correlation coefficient (MCC), as it has been shown to be a better evaluation metric for binary classification tasks, especially when dealing with class imbalances (Chicco and Jurman 2020). We also report the area under the precision-recall curve (AUPRC), further evaluation metrics are listed in the supplement (Table ST1, available at supplementary material  *Bioinformatics Advances* online). Table 2 details the median performances across all folds of both the sequence-based predictions (Sequences) and the combined approaches (Sequences + Features). For ClinIAN, we pre-trained TabNet within each CV fold on the prediction task and initialized ClinIAN with these learned weights.

**Table 2:** Comparison of the sequence-based mutation model (ClinIAN_seq_) and ClinIAN against RF^a^, LinSVM^b^, PolySVM^c^, and the state-of-the-art method MOMA (Moon and Lee 2022).

	MCC [CI]	AUPRC [CI]
	**Mutations**	
**RF**	**0.14** [0.07, 0.48]	**0.13** [0.11, 0.38]
**LinSVM**	**0.14** [0.07, 0.49]	**0.13** [0.11, 0.38]
**PolySVM**	**0.14** [0.07, 0.49]	**0.13** [0.11, 0.38]
**ClinIAN_seq_**	**0.14** [0.07, 0.46]	0.12 [0.11, 0.35]
**ClinIAN_seq; prott5_**	0.05 [-0.02, 0.29]	0.11 [0.05, 0.26]
**ClinIAN**	–	–
**MOMA**	–	–
**ClinIAN_prott5_**	–	–
	**Mutations + Metadata**	
**RF**	0.16 [0.09, 0.48]	0.14 [0.11, 0.37]
**LinSVM**	0.14 [0.05, 0.38]	0.13 [0.09, 0.29]
**PolySVM**	0.14 [0.05, 0.37]	0.13 [0.09, 0.28]
**ClinIAN_seq_**	–	–
**ClinIAN_seq; prott5_**	–	–
**ClinIAN**	0.18 [0.10, 0.28]	0.14 [0.12, 0.20]
**MOMA**	0.05 [0.02, 0.14]	0.11 [0.10, 0.13]
**ClinIAN_prott5_**	**0.20** [0.09, 0.27]	**0.15** [0.12, 0.18]

We further evaluated ClinIAN’s ability to incorporate more complex pLM embeddings, using embeddings from prott5-xl-uniref50 (Elnaggar *et al.* 2021). Performances are reported as the median AUPRC^d^ and MCC^e^, with confidence intervals (CIs), across the 10 × 10 nested CV iterations. ClinIAN and MOMA were only applied to the combined data, and ClinIAN_seq_ was only applied to mutations.

a Random Forest.

b Linear SVM.

c Polynomial SVM.

d Area under the precision-recall curve.

e Matthews correlation coefficient.

### 3.1 Implementation

We implemented ClinIAN in Python using Pytorch (Paszke *et al.* 2019) (v2.5.1). The baseline models and pipeline utilities were implemented in Scikit-learn (v1.7.1) (Pedregosa *et al.* 2011). All models used the same training loop, implemented with skorch (Tietz *et al.* 2017), which provides an interface between PyTorch and Scikit-learn. We trained the PyTorch models with early stopping and a maximum of 200 epochs using the Adam optimizer (Kingma 2014). We used Pango Aliasor (v0.3.0) for the variant mapping. The code was executed on a single Nvidia GeForce GTX 1080 Ti GPU.

### 3.2 Quantifying classification performances

First, we benchmarked ClinIAN_seq_ and the baselines using only sequences. These results are shown in the upper part of Table 2. We observed difficulties in predicting mortality from the sequences without additional information across all models. The median performances were low, with considerable confidence intervals (CI). We report the median, which is more robust against extreme outliers than the mean. These performances confirm the assumption that many other factors contribute to the progression of COVID-19. Therefore, it is preferable to combine the viral mutations with clinical parameters.

In the lower part of Table 2 are the performance measures of ClinIAN against the baseline models on the combined features set. We observed that simply combining features did not improve the performance of SVM. While RF performed better with the combined features, ClinIAN outperformed the baselines in MCC and AUPRC. Additionally, ClinIAN exhibited greater performance stability than the other models, except for the state-of-the-art method MOMA, indicated by the smaller confidence interval. While the state-of-the-art method MOMA exhibited greater stability, it did not perform as expected. This suggests that in a prediction setting such as COVID-19, where numerous factors influence disease outcomes, it is beneficial to integrate both clinical metadata and mutations. ClinIAN’s performance could be further improved by utilizing the protein language model (pLM) embeddings from prott5-xl-uniref50 (Elnaggar *et al.* 2021) (ClinIAN_prott5_). The observed increase in performance, while not statistically significant (Sec. S3.1, available at supplementary material  *Bioinformatics Advances* online), suggests a more stable learning process.

### 3.3 Identifying predictive features

The above comparative analysis demonstrated that ClinIAN exhibits greater performance stability than baseline approaches when addressing this challenging predictive task. Our approach outperforms the state-of-the-art method MOMA, although MOMA shows slightly more stability in terms of SD. A strong advantage of ClinIAN is its ability to derive insights directly from its predictions, thereby circumventing the need for post hoc explanations by integrating MIL attention layers.

We visualized the feature importances (FI) by plotting the distance between each feature’s observed (*a*) and expected attention (*E[a]*), with FI=a−E[a]. We computed the expected attention for each sample assuming a uniform distribution, then averaged it across the entire dataset. Consequently, mutation positions and clinical features that were more frequently observed were allocated higher expected attention values (see supplement Sec. S3.2, available at supplementary material  *Bioinformatics Advances* online). Mutation positions in proteins with more mutations than other proteins therefore received a lower expected attention due to the assumption of a uniform distribution. Additionally, we analyzed the FIs for both protein and clinical parameters. These FI maps facilitate a systematic exploration of our model, starting from the output layer and working backward to the individual features. We can use these FI maps to identify protein and mutation-level associations with the outcome.

**Figure 2:**
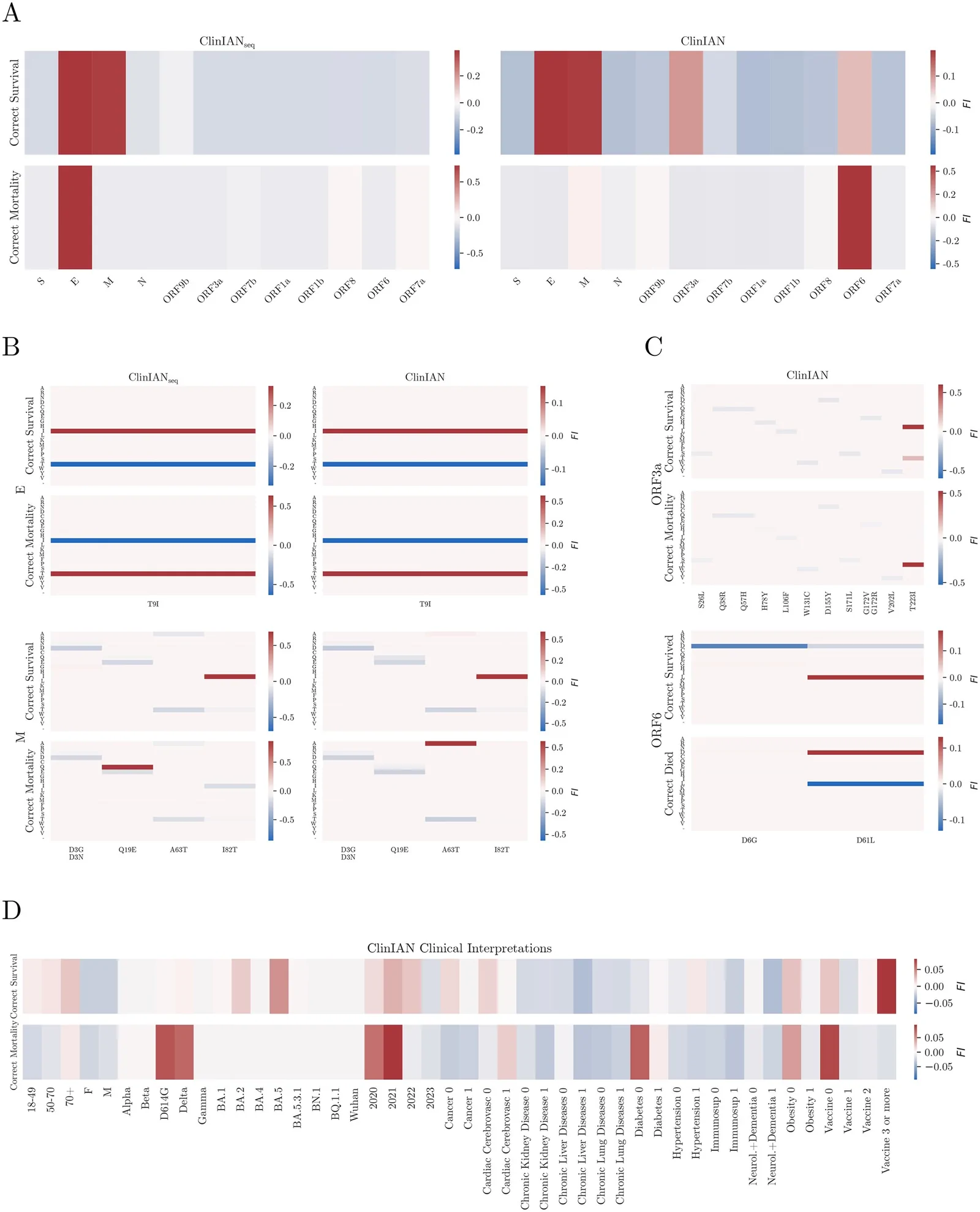
Visualization of FI masks of ClinIAN and ClinIAN_seq_ across the subset of correctly classified samples. (A) Compares the identified FI across proteins. ClinIAN identifies more relevant proteins for host survival. (B) Shows the comparison of both model interpretations (left: ClinIAN_seq_; right: ClinIAN) on the envelope (E) and membrane (M) protein. (C) Visualizes ORF3a and ORF6 attentions of ClinIAN. (D) shows identified clinical features of ClinIAN, emphasizing the number of vaccinations as an important predictive feature.

Figure 2A displays FI maps from ClinIAN_seq_ and ClinIAN highlighting the disparities in the proteins that are deemed significant by each model. We categorize the correctly classified samples into two groups: “survival” and “mortality.” ClinIAN_seq_ places considerable emphasis on the envelope protein (E) for both groups. Additionally, it assigns the membrane protein M great power in predicting survival. ClinIAN similarly assigns the M protein greater power in predicting survival, while also underscoring the significance of ORF3a and ORF6 in classifying survivability. Notably, ClinIAN places greater emphasis on ORF6 for patients who did not survive, diverging from ClinIAN_seq’_s focus.

For both models, we could dive deeper into decision-making by evaluating the FIs assigned to each mutation position. Figure 2B provides a comparative visualization of E and M for ClinIAN (right) and ClinIAN_seq_ (left), focusing on patients accurately classified as survivors. The envelope protein E only exhibits one mutation, *T9I*. We observed a consistency within FI, with the original amino acid *T* being important for predicting patients who passed away, and the substitution to *I* being weighted highly for surviving patients. The mutation *T9I* occurred between November 2021 and January 2022 and is indicative of Omicron variants. This specific variant has been associated to lower virulence (Xia *et al.* 2022). In both models, the membrane protein M receives significant FIs among surviving patients. In contrast, it is only weighted highly in patients who died by ClinIAN_seq_. Both mutations *Q19E* and *A63T* are defining mutations for the Omicron variants (Hossain *et al.* 2022). *I82T*, with the original amino acid I being associated with host survival, has been identified prior as a minor mutation in the Delta variant, while the membrane protein, due to its low variability, has been suggested as a possible target for antibody development (Zhang *et al.* 2022).

Figure 2C displays additional interpretations of ClinIAN for the identified proteins ORF3a and ORF6. The mutation *T223I* is associated with host survival, while the original amino acid seems to be associated with mortality. *T223I* has occured in Omicron variants, starting with BA.2 (Wang *et al.* 2023). ClinIAN also pinpoints the mutation at position 61 in ORF6 as important for identifying patients who passed away, with the original amino acid *D* predictive of mortality. The mutation *D61L* has been identified in the Omicron variants BA.2 and BA.4 and subvariants, as well as some recombinants (BN.1) (Hossain *et al.* 2022).

Figure 2D displays the TabNet interpretations of ClinIAN for the same two cohorts. Our analysis indicates that receiving three or more vaccinations (“Vaccine 3 or more”) is a strong predictor of host survival. Conversely, individuals with zero vaccinations (“Vaccine 0”) are associated with increased mortality. Additionally, the variants *D614G* and *Delta*, alongside the years of patient’admission to the hospital 2020 and 2021, seem to be highly predictive of mortality. This is consistent, as both variants occurred in 2020 and 2021. Surprisingly, the absence of comorbidities, such as “no obesity” and “no diabetes,” is predictive of mortality within our cohort. Indeed, within our data, patients without these comorbidities tended to be less vaccinated than patients with these comorbidities. Along with prioritization in the vaccine distribution, one explanation could be a false sense of security among people without these comorbidities, leading them not to take the vaccine. This finding is consistent with the mean vaccination level among patients who passed away, which was 0.41 for patients without diabetes and 0.63 for patients with diabetes. Similar values were observed for obese patients.

In addition to these findings, we validated the significance of the identified mutations on viral sequences shared via GISAID, the global data science initiative (Khare *et al.* 2021). All mutations discussed above show a highly significant association with the outcome, which validates the interpretative value of our proposed model. Details can be found in the supplementary materials Sec. S3.7, available at supplementary material  *Bioinformatics Advances* online. We further validated that these correlations are indeed positive associations with the corresponding outcome label (see supplement Sec. S3.3, available at supplementary material  *Bioinformatics Advances* online).

In summary, our analysis identified several non-spike mutations that emerged in late 2021 and early 2022 and are typical of certain Omicron lineages and sublineages. Notably, these mutations are associated with survival in our models, corroborating existing knowledge of the lower virulence of Omicron variants.

## 4 Conclusion

In this study, we introduce ClinIAN, an innovative and inherently interpretable end-to-end learning model that effectively combines clinical metadata and viral sequences. Our evaluation of ClinIAN underscores its potential in addressing the complex challenge of predicting COVID-19 disease severity by integrating viral sequence mutations with clinical features, an area typically associated with limited predictive performance. Our findings indicate that ClinIAN not only surpasses established baseline methods but also outperforms the contemporary state-of-the-art approach, MOMA. This demonstrates the model’s capacity to effectively leverage information from multiple data modalities, enhancing predictive accuracy.

Moreover, the versatility of ClinIAN suggests its applicability extends beyond COVID-19, with potential utility in studying other viral diseases and parasitic conditions, such as malaria, as well as in cancer research. By integrating clinical insights with genetic data from pathogens or cancerous cells, ClinIAN opens new avenues for exploring disease mechanisms and outcomes. However, applicability in these other contexts would first require thorough evaluation and is only assumed, as the main limitations are the number of data points and the availability of tabular data with matching sequence data rather than the underlying data itself.

Importantly, ClinIAN offers inherent interpretability of its decision processes across various hierarchical levels, highlighting the influence of specific proteins and mutation positions. The integration with TabNet adds a layer of interpretability and a way to correct for biases in mutation positions by utilizing expressive embeddings. This combination of modalities provides a deeper understanding of clinical features. Our decision-making analysis also confirmed the biological relevance of the interpretations provided by ClinIAN, thus highlighting it as a promising explorative tool in medical research.

We acknowledge the limitations within our analysis. ClinIAN in its current form can not deal with missing data and will be further explored in the future. Excluding features with high missingness can limit the clinical applicability of our analysis. This was done to remove biases introduced by different imputation strategies, as our main objective was not to maximize performance but to provide a template for what an inherently interpretable model merging tabular clinical data and viral sequence data can look like while still delivering state-of-the-art performance. Introducing biases through imputation complicates this interpretation and requires a careful approach (Shadbahr *et al.* 2023). Nonetheless, we acknowledge that the assumption of no missing data does not necessarily hold true in clinical applications. Further, the complexity of predicting mortality, as reflected in the modest current absolute performance, makes its application in a clinical setting difficult. Still, combining tabular clinical features with viral sequences like this is a step in the right direction.

## Supplementary Material

vbag254_Supplementary_Data

## Data Availability

Due to the sensitive nature of patient data, which is protected under GDPR and additional federal laws, we cannot make the data avilable openly. Nevertheless, data can be requested at the EuCARE data access committee: https://eucareresearch.eu/eucare-database/. Code is available on https://github.com/pfeiferAI/ClinIAN and on Zenodo (DOI: 10.5281/zenodo.17177451).

## References

[vbag254-B1] Abdullah F , Myers J, Basu D et al Decreased severity of disease during the first global omicron variant covid-19 outbreak in a large hospital in tshwane, South Africa. Int J Infect Dis 2022;116:38–42. 10.1016/j.ijid.2021.12.357 PMC8713416 34971823

[vbag254-B2] Aksamentov I , Roemer C, Hodcroft EB et al Nextclade: clade assignment, mutation calling and quality control for viral genomes. JOSS 2021;6:3773. 10.21105/joss.03773

[vbag254-B3] Arik SÖ , Pfister T. TabNet: attentive interpretable tabular learning. AAAI 2021;35:6679–87. 10.1609/aaai.v35i8.16826

[vbag254-B4] Bai F , Santoro A, Hedberg P et al The omicron variant is associated with a reduced risk of the post covid-19 condition and its main phenotypes compared to the wild-type virus: results from the EuCARE-POSTCOVID-19 study. Viruses 2024;16:1500. 10.3390/v16091500 PMC11437468 39339976

[vbag254-B5] Balcı AT , Ebeid MM, Benos PV et al An intrinsically interpretable neural network architecture for sequence-to-function learning. Bioinformatics 2023;39:i413–22. 10.1093/bioinformatics/btad271 PMC10311317 37387140

[vbag254-B6] Bellerba F , Bardeck N, Boehm M et al SARS-CoV-2 trends in Italy, Germany and Portugal and school opening during the period of omicron variant dominance: a quasi experimental study in the eucare project. Int J Infect Dis 2024;138:63–72. 10.1016/j.ijid.2023.11.002 37956899

[vbag254-B7] Bordt S , Finck M, Raidl E et al Post-hoc explanations fail to achieve their purpose in adversarial contexts. In: *Proceedings of the 2022 ACM Conference on Fairness, Accountability, and Transparency*, p. 891–905, New York, NY, USA. Association for Computing Machinery, 2022. 10.1145/3531146.3533153

[vbag254-B8] Borisov V , Leemann T, Seßler K et al Deep neural networks and tabular data: a survey. IEEE Trans Neural Netw Learn Syst 2024;35:7499–519. 10.1109/TNNLS.2022.3229161 37015381

[vbag254-B10] Carbonneau M-A , Cheplygina V, Granger E et al Multiple instance learning: a survey of problem characteristics and applications. Pattern Recognit 2018;77:329–53. 10.1016/j.patcog.2017.10.009

[vbag254-B11] Chicco D , Jurman G. The advantages of the Matthews correlation coefficient (MCC) over F1 score and accuracy in binary classification evaluation. BMC Genomics 2020;21:6. 10.1186/s12864-019-6413-7 PMC6941312 31898477

[vbag254-B12] Cui Y , Jia M, Lin T-Y et al Class-balanced loss based on effective number of samples. In: *Proceedings of the IEEE/CVF Conference on Computer Vision and Pattern Recognition*, p. 9268–77, Long Beach, CA, USA. IEEE, 2019. 10.1109/CVPR.2019.00949

[vbag254-B13] Dey L , Chakraborty S, Mukhopadhyay A. Machine learning techniques for sequence-based prediction of viral–host interactions between SARS-CoV-2 and human proteins. Biomed J 2020;43:438–50. 10.1016/j.bj.2020.08.003 PMC7470713 33036956

[vbag254-B14] Dietterich TG , Lathrop RH, Lozano-Pérez T. Solving the multiple instance problem with axis-parallel rectangles. Artif Intell 1997;89:31–71. 10.1016/S0004-3702(96)00034-3

[vbag254-B15] Ditz JC , Reuter B, Pfeifer N. Comic: convolutional kernel networks for interpretable end-to-end learning on (multi-) omics data. Bioinformatics 2023a;39:i76–85. 10.1093/bioinformatics/btad204 PMC10311322 37387152

[vbag254-B16] Ditz JC , Reuter B, Pfeifer N. Inherently interpretable position-aware convolutional motif kernel networks for biological sequencing data. Sci Rep 2023b;13:17216. 10.1038/s41598-023-44175-7 PMC10567796 37821530

[vbag254-B17] Do C. , Lähdesmäki H. Incorporating hierarchical information into multiple instance learning for patient phenotype prediction with single-cell RNA-sequencing data. Bioinformatics 2025;41:i96–104. 10.1093/bioinformatics/btaf241 PMC12261414 40662783

[vbag254-B18] Elnaggar A , Heinzinger M, Dallago C et al Prottrans: towards cracking the language of life’s code through self-supervised learning. IEEE Trans Pattern Anal Mach Intell 2021;44:7112–27. 10.1109/TPAMI.2021.3095381 34232869

[vbag254-B19] Hake A , Germann A, de Beer C et al Insights to HIV-1 coreceptor usage by estimating HLA adaptation with Bayesian generalized linear mixed models. PLoS Comput Biol 2023;19:e1010355. 10.1371/journal.pcbi.1010355 PMC10769057 38127856

[vbag254-B20] Hedberg P , Varisco B, Bai F et al Eucare-hospitalised study protocol: a cohort study of patients hospitalised with covid-19 in the eucare project. BMC Infect Dis 2023;23:690. 10.1186/s12879-023-08658-2 PMC10580565 37845624

[vbag254-B21] Hedberg P , Parczewski M, Serwin K et al In-hospital mortality during the wild-type, alpha, delta, and omicron SARS-CoV-2 waves: a multinational cohort study in the eucare project. The Lancet Regional Health–Europe 2024;38:100855. 10.1016/j.lanepe.2024.100855 PMC10928271 38476753

[vbag254-B22] Hoffman GE. Correcting for population structure and kinship using the linear mixed model: theory and extensions. PLoS One 2013;8:e75707. 10.1371/journal.pone.0075707 PMC3810480 24204578

[vbag254-B23] Hossain A , Akter S, Rashid AA et al Unique mutations in SARS-CoV-2 omicron subvariants’ non-spike proteins: potential impacts on viral pathogenesis and host immune evasion. Microb Pathog 2022;170:105699. 10.1016/j.micpath.2022.105699 PMC9356572 35944840

[vbag254-B24] Ilse M , Tomczak J, Welling M. Attention-based deep multiple instance learning. In: International Conference on Machine Learning, p. 2127–36. PMLR, 2018. 10.48550/arXiv.1802.04712; https://proceedings.mlr.press/v80/ilse18a.html, preprint: not peer reviewed.

[vbag254-B25] Jumper J , Evans R, Pritzel A et al Highly accurate protein structure prediction with alphafold. Nature 2021;596:583–9. 10.1038/s41586-021-03819-2 PMC8371605 34265844

[vbag254-B26] Kehrer T , Cupic A, Ye C et al Impact of SARS-CoV-2 orf6 and its variant polymorphisms on host responses and viral pathogenesis. Cell Host Microbe 2023;31:1668–84.e12. 10.1016/j.chom.2023.08.003 PMC10750313 37738983

[vbag254-B27] Khare S , Gurry C, Freitas L et al Gisaid’s role in pandemic response. China CDC Wkly 2021;3:1049–51. 10.46234/ccdcw2021.255 PMC8668406 34934514

[vbag254-B28] Kingma DP. Adam: A method for stochastic optimization. In: *Proc. International Conference on Learning Representations*. ICLR, 2015. 10.48550/arXiv.1412.6980

[vbag254-B29] Lundberg SM , Lee S-I. A unified approach to interpreting model predictions. In: Guyon I, Luxburg UV, Bengio S, Wallach H, Fergus R, Vishwanathan S, Garnett R (eds.), Advances in Neural Information Processing Systems, Vol. 30. Red Hook, NY, USA: Curran Associates, Inc., 2017. 10.48550/arXiv.1705.07874; https://proceedings.neurips.cc/paper_files/paper/2017/file/8a20a8621978632d76c43dfd28b67767-Paper.pdf

[vbag254-B30] MacEachern SJ , Forkert ND. Machine learning for precision medicine. Genome 2021;64:416–25. 10.1139/gen-2020-0131 33091314

[vbag254-B31] McElfresh D , Khandagale S, Valverde J et al When do neural nets outperform boosted trees on tabular data? In: Oh A, Naumann T, Globerson A, Saenko K, Hardt M, Levine S (eds.), Advances in Neural Information Processing Systems, Vol. 36, p. 76336–69. Red Hook, NY, USA: Curran Associates, Inc., 2023. 10.48550/arXiv.2305.02997; https://proceedings.neurips.cc/paper_files/paper/2023/file/f06d5ebd4ff40b40dd97e30cee632123-Paper-Datasets_and_Benchmarks.pdf

[vbag254-B32] Mohammed I , Nauman A, Paul P et al The efficacy and effectiveness of the covid-19 vaccines in reducing infection, severity, hospitalization, and mortality: a systematic review. Hum Vaccin Immunother 2022;18:2027160. 10.1080/21645515.2022.2027160 PMC8862168 35113777

[vbag254-B33] Moon S , Lee H. MOMA: a multi-task attention learning algorithm for multi-omics data interpretation and classification. Bioinformatics 2022;38:2287–96. 10.1093/bioinformatics/btac080 PMC10060719 35157023

[vbag254-B34] Nicola M , Alsafi Z, Sohrabi C et al The socio-economic implications of the coronavirus pandemic (COVID-19): a review. Int J Surg 2020;78:185–93. 10.1016/j.ijsu.2020.04.018 PMC7162753 32305533

[vbag254-B35] O’Toole Á , Scher E, Underwood A et al Assignment of epidemiological lineages in an emerging pandemic using the pangolin tool. Virus Evol 2021;7:veab064. 10.1093/ve/veab064 PMC8344591 34527285

[vbag254-B36] Paszke A , Gross S, Massa F et al PyTorch: an imperative style, high-performance deep learning library. Advances in Neural Information Processing Systems, Vol. 32, p. 8026-37. Curran Associates, Inc., Red Hook, NY, USA, 2019. 10.48550/arXiv.1912.01703; https://proceedings.neurips.cc/paper/2019/hash/bdbca288fee7f92f2bfa9f7012727740-Abstract.html

[vbag254-B37] Pedregosa F , Varoquaux G, Gramfort A et al Scikit-learn: machine learning in Python. The Journal of Machine Learning Research 2011;12:2825–30.

[vbag254-B38] Rambaut A , Holmes EC, O’Toole Á et al A dynamic nomenclature proposal for SARS-CoV-2 lineages to assist genomic epidemiology. Nat Microbiol 2020;5:1403–7. 10.1038/s41564-020-0770-5 PMC7610519 32669681

[vbag254-B39] Rudin C. Stop explaining black box machine learning models for high stakes decisions and use interpretable models instead. Nat Mach Intell 2019;1:206–15. 10.1038/s42256-019-0048-x PMC9122117 35603010

[vbag254-B40] Sarvamangala D , Kulkarni RV. Convolutional neural networks in medical image understanding: a survey. Evol Intell 2022;15:1–22. 10.1007/s12065-020-00540-3 PMC7778711 33425040

[vbag254-B41] Seabra SG , Merca F, Pereira B et al Serological screening in a large-scale municipal survey in cascais, Portugal, during the first waves of the covid-19 pandemic: lessons for future pandemic preparedness efforts. Front Public Health 2024;12:1326125. 10.3389/fpubh.2024.1326125 PMC10869482 38371240

[vbag254-B42] Shadbahr T , Roberts M, Stanczuk J et al The impact of imputation quality on machine learning classifiers for datasets with missing values. Commun Med (Lond) 2023;3:139. 10.1038/s43856-023-00356-z PMC10558448 37803172

[vbag254-B43] Shrikumar A , Greenside P, Kundaje A. Learning important features through propagating activation differences. In: Precup The D YW (eds.), Proceedings of the 34th International Conference on Machine Learning, Volume 70 of Proceedings of Machine Learning Research, p. 3145–53. PMLR, 2017. 10.48550/arXiv.1704.02685; https://proceedings.mlr.press/v70/shrikumar17a

[vbag254-B44] Tietz M , Fan TJ, Nouri D et al skorch: A scikit-learn compatible neural network library that wraps PyTorch. https://skorch.readthedocs.io/en/stable/ (July 2017)

[vbag254-B45] Walia K , Sharma A, Paul S et al SARS-CoV-2 virulence factor ORF3a blocks lysosome function by modulating TBC1D5-dependent Rab7 GTPase cycle. Nat Commun 2024;15:2053. 10.1038/s41467-024-46417-2 PMC10918171 38448435

[vbag254-B46] Wang W , Qu Y, Wang X et al Genetic variety of ORF3a shapes SARS-CoV-2 fitness through modulation of lipid droplet. J Med Virol 2023;95:e28630. 10.1002/jmv.28630 36861654

[vbag254-B47] Wang X , Yan Y, Tang P et al Revisiting multiple instance neural networks. Pattern Recognit 2018;74:15–24. 10.1016/j.patcog.2017.08.026

[vbag254-B48] WHO. Covid-19 epidemiological update. https://www.who.int/publications/m/item/covid-19-epidemiological-update—24-december-2024, 2024.

[vbag254-B49] Xia B , Wang Y, Pan X et al Why is the SARS-CoV-2 omicron variant milder? Innovation 2022;3:100251. 10.1016/j.xinn.2022.100251 PMC9040510 35497020

[vbag254-B50] Xiang Y , Jia Y, Chen L et al COVID-19 epidemic prediction and the impact of public health interventions: a review of COVID-19 epidemic models. Infect Dis Model 2021;6:324–42. 10.1016/j.idm.2021.01.001 PMC7790451 33437897

[vbag254-B51] Zhang Z , Nomura N, Muramoto Y et al Structure of SARS-CoV-2 membrane protein essential for virus assembly. Nat Commun 2022;13:4399. 10.1038/s41467-022-32019-3 PMC9355944 35931673

