# Supplementary material for: ClinIAN: Clinically Informed Attention Network
Source: Bioinform Adv. 2026 Sep 9;6(1):vbag254. doi: 10.1093/bioadv/vbag254 (PMC13596770; doi:10.1093/bioadv/vbag254)
Supplement: Supplementary file 1 [file vbag254_supplementary_data.pdf]

ClinIAN: Clinically Informed Attention Network for disease severity prediction in COVID-19, an EuCARE study
Supplementary Materials

Contents

S1 EuCARE Whole Genome Sequencing Cohort **3**
S1.1 Data Preparation 4
S1.2 Sensitivity Analysis of quality control thresholds 5
S1.3 Correlation Analysis 7

S2 ClinIAN - Clinically Informed Attention Network **9**
S2.1 Utilizing Mutation Data 9
S2.2 Combining Sequence Data with Clinical Information 10

S3 Predicting COVID-19 mortality **10**
S3.1 Quantifying classification performances 13
S3.2 Identifying predictive features 15
S3.3 Directionality Analysis 20
S3.4 Ablation Study: Clinical vs. Sequence vs. Combined 23
S3.5 Ablation Study: Model Performance without VOC and calendar year . . 23
S3.6 Training Setup: ClinIAN 25
S3.7 Statistical Validation 26
S3.8 MOMA - A Multi-task Attention Learning Algorithm for Multi-omics Data Interpretation and Classification 29

S4 Reproducibility **29**

List of Tables

ST1 Performance Comparison 14
ST2 Ablation: Sequence vs. Feature vs. Combined 23
ST3 Ablation: No VOC and Calendar Year 23
ST4 Statistical Validation: GISAID 26

List of Figures

SF1 Feature distributions: Mortality . . . 3
SF2 Feature distributions: Time . . . 4
SF3 Preprocessing . . . 5
SF4 Sensitivity Analysis for Mutation threshold . . . 5
SF5 Correlation Analysis . . . 7
SF6 Visualization of the proposed models . . . 9
SF7 Protein Interpretations . . . 16
SF8 E, M Interpretations . . . 17
SF9 ORF3a, ORF6 Interpretations . . . 18
SF10 Clinical Interpretations . . . 19
SF11 Directionalities: Proteins . . . 21
SF12 Directionalities: Envelope Protein . . . 21
SF13 Directionalities: Membrane Protein . . . 22
SF14 Directionalities: ORF3a Protein . . . 22
SF15 Directionalities: ORF6 Protein . . . 22
SF16 ClinIAN: Validation MCC Training Curve . . . 25

S1 EuCARE Whole Genome Sequencing Cohort

We present a detailed overview of all features used in the analysis stratified by the label mortality Figure SF1. And distributed across time Figure SF2.

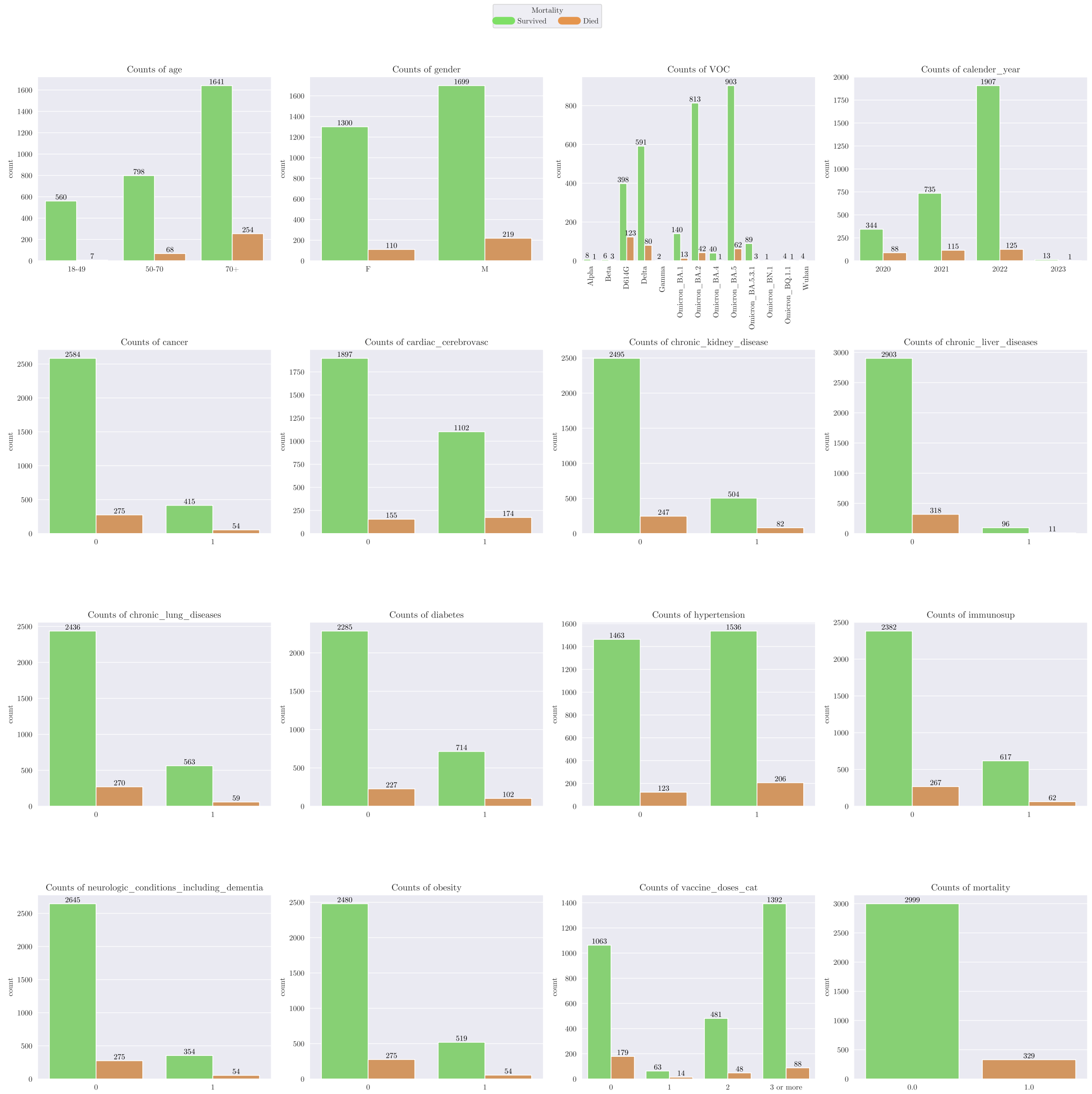

Figure SF1: Feature distributions stratified by mortality. Also visualized is the class imbalance in mortality.

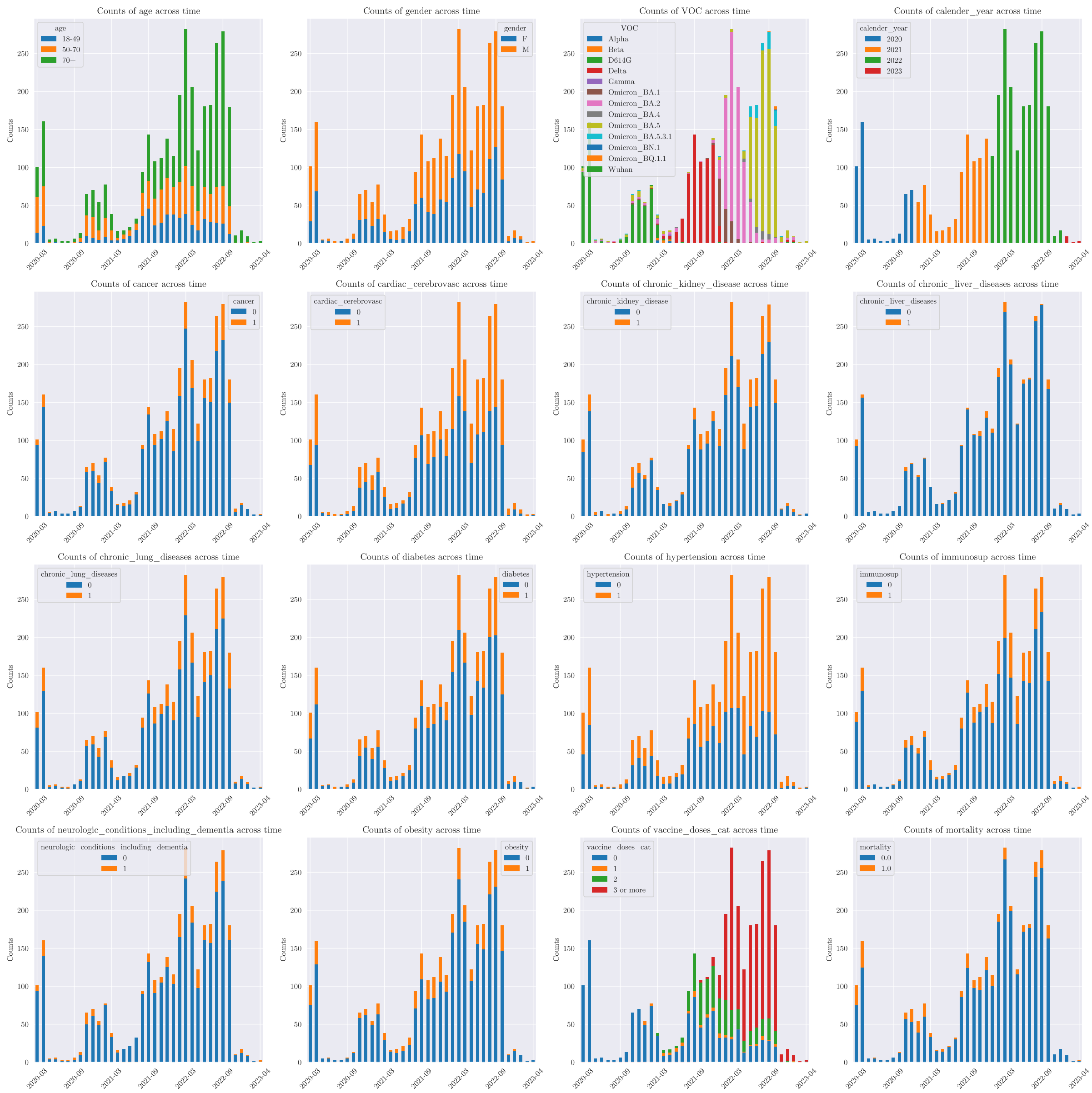

Figure SF2: Feature distributions across time

S1.1 Data Preparation

We present more details about the processing steps of producing the final dataset used in the evaluations. Figure SF10 displays individual initial samples get removed as they cannot be aligned to the reference sequence. Further, 2477 samples are removed due to quality definitions. We only want to use sequences of high enough quality. Therefore, we remove sequences with more than 10% ambiguous residues ("X"), as they provide no information. Several sequences must be removed as they are much shorter than expected. We apply a conservative threshold, allowing all sequences that are at least 80% of the reference sequence length. We exclude sequences for patients with multiple sequences (477). Further exclusions occur for 617 samples with stop codons in the middle of proteins. Finally, we have to remove 63 samples that lack vaccination information.

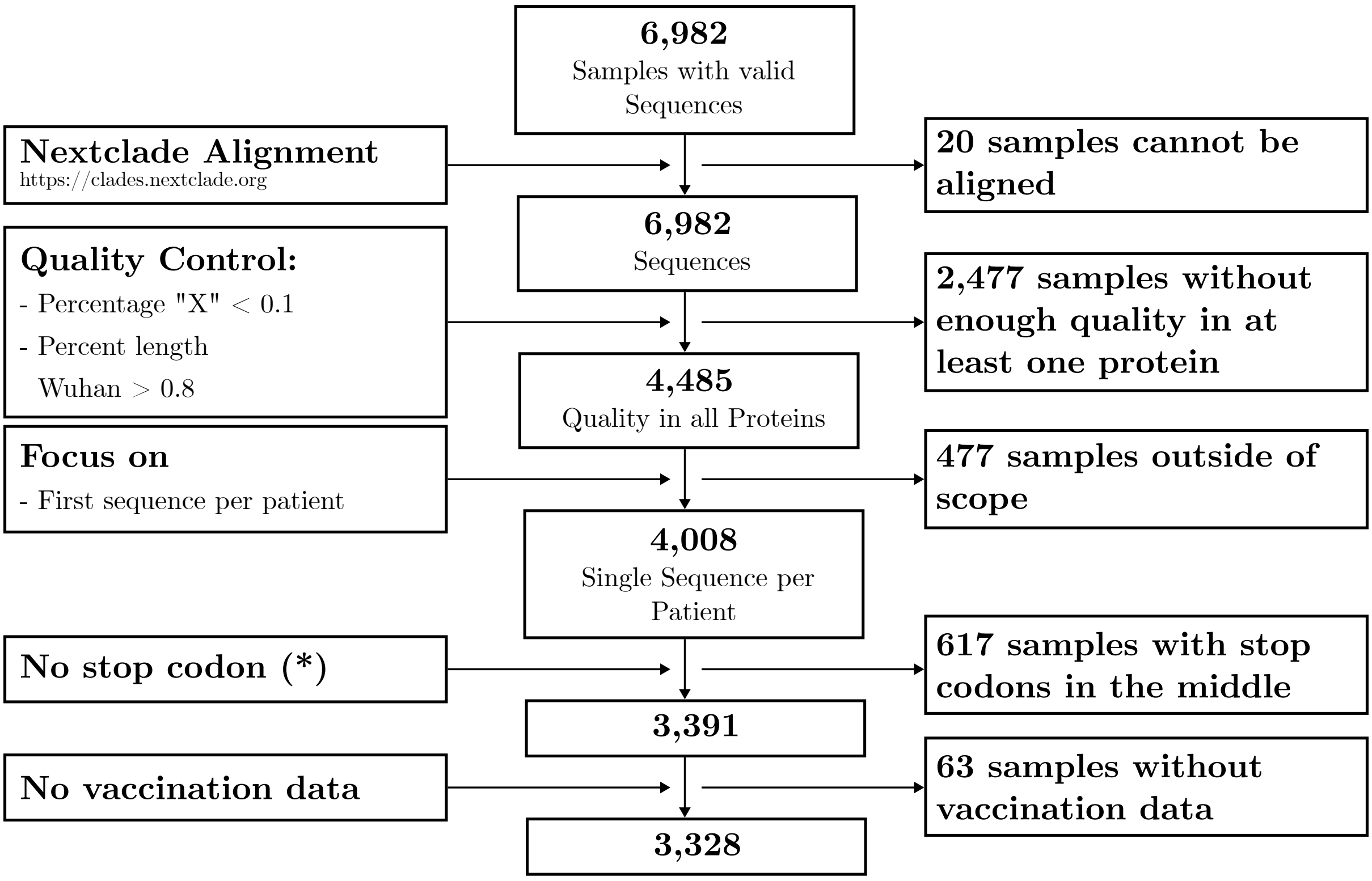

Figure SF3: Detailed steps about the preprocessing from 6,982 samples in the database to the final dataset of 3,328 patients.

S1.2 Sensitivity Analysis of quality control thresholds

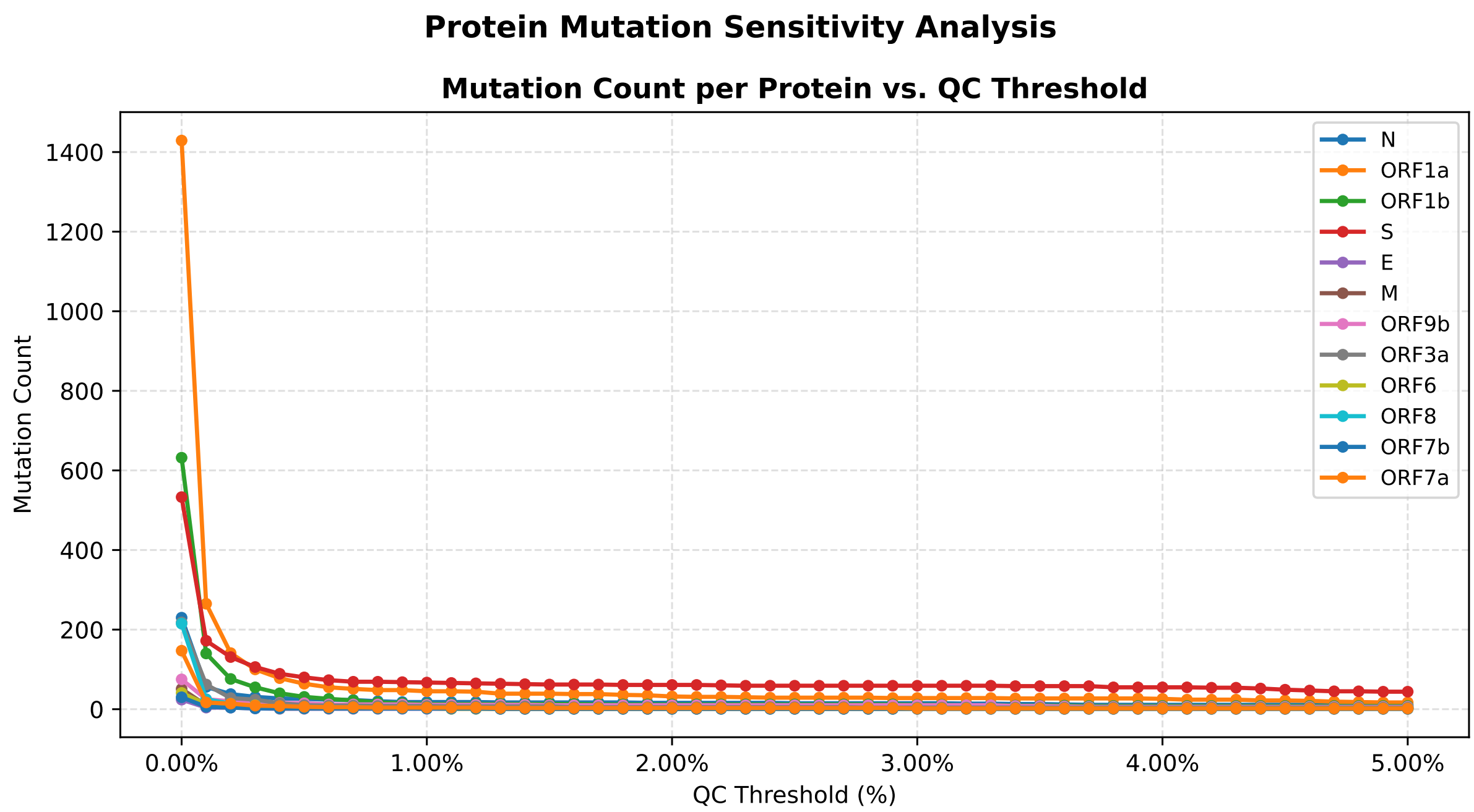

Figure SF4: Line-plot depicting the absolute number of mutations per protein depending on the different levels of the exclusion threshold for mutations.

We provide a brief sensitivity analysis for the quality control threshold of 0.5%, the

minimum frequency of occurrence of a single mutation considered in our analysis. In Figure SF4, we depict differing levels of thresholds applied and the effects on the number of samples per protein. We visualized the range from 0% (all mutations considered) to 5% in increments of 0.1%. It becomes clear that there is a steep drop-off in the number of mutations, especially for the ORF1a, ORF1b, and Spike proteins, within the first step. This implies that a large number of mutations occur no more than 0.1% of the time. In absolute numbers, when projected across the entire dataset, this means only up to 3 samples contain these mutations. Another larger step is from 0.1% to 0.2%, and afterward, fewer and fewer mutations are filtered out by this step.

The threshold of 0.5% (>15 samples) was applied because low-frequency binary features provide too few observations to support a stable, and more importantly, interpretable estimate of their association with the outcome. In the 10x10 nested cross-validation setting, such rare features additionally risk being absent or near-constant within individual training folds.

S1.3 Correlation Analysis

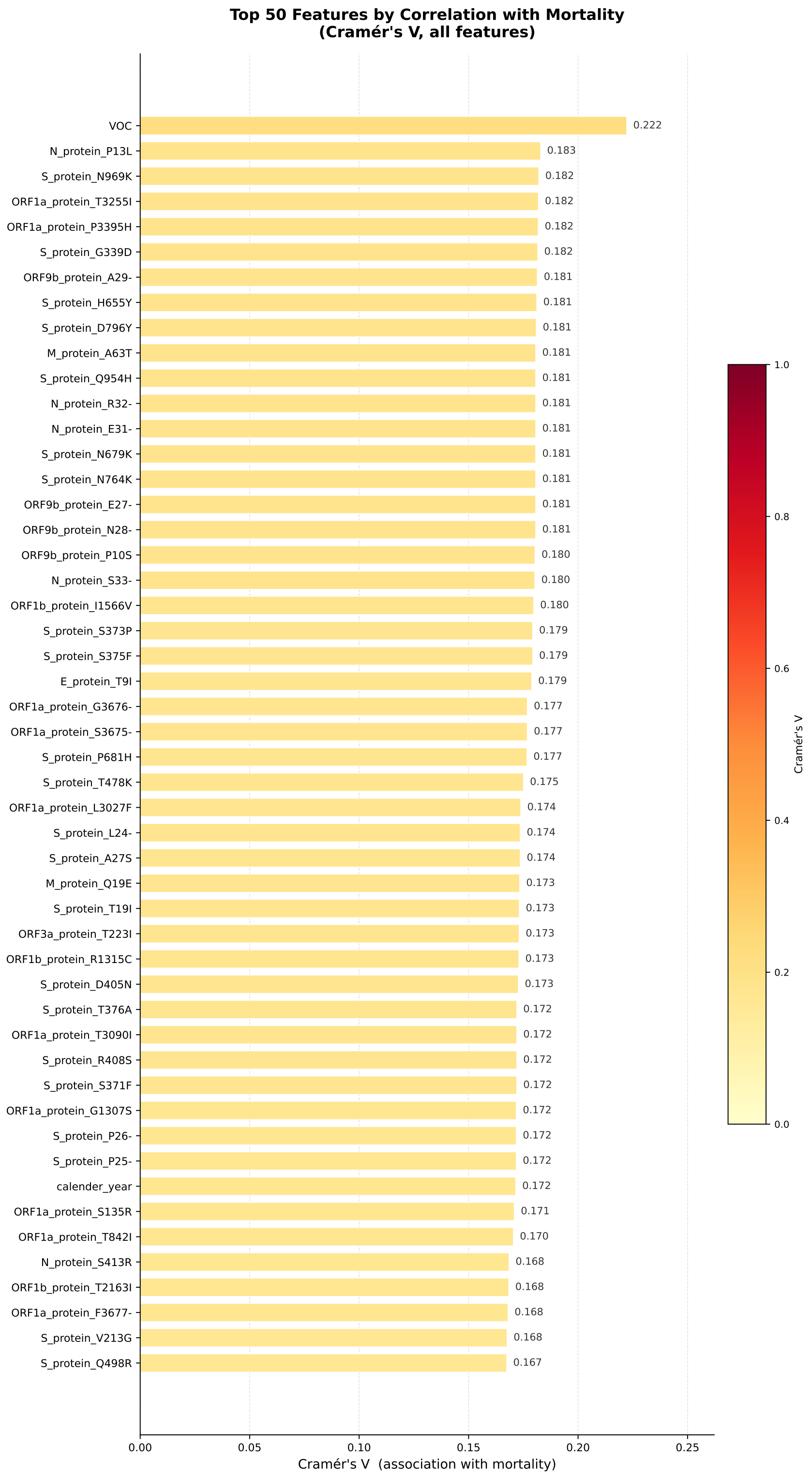

Figure SF5: Barchart depicting the one-by-one correlations between the top-50 features and the outcome label mortality.

We provide an analysis of the correlations between the label mortality and the top 50 features used in the analysis. We visualize the Cramer‘s V correlation. The analysis indicates that no single feature explains the outcome. The VOC shows the strongest

correlation, with some variant-defining mutations among the top correlations. While we find most of the highlighted mutations within the paper among the top 50 features, our identified ORF6:D61L mutation does not occur within this most correlated features.

S2 ClinIAN - Clinically Informed Attention Network

S2.1 Utilizing Mutation Data

A

B

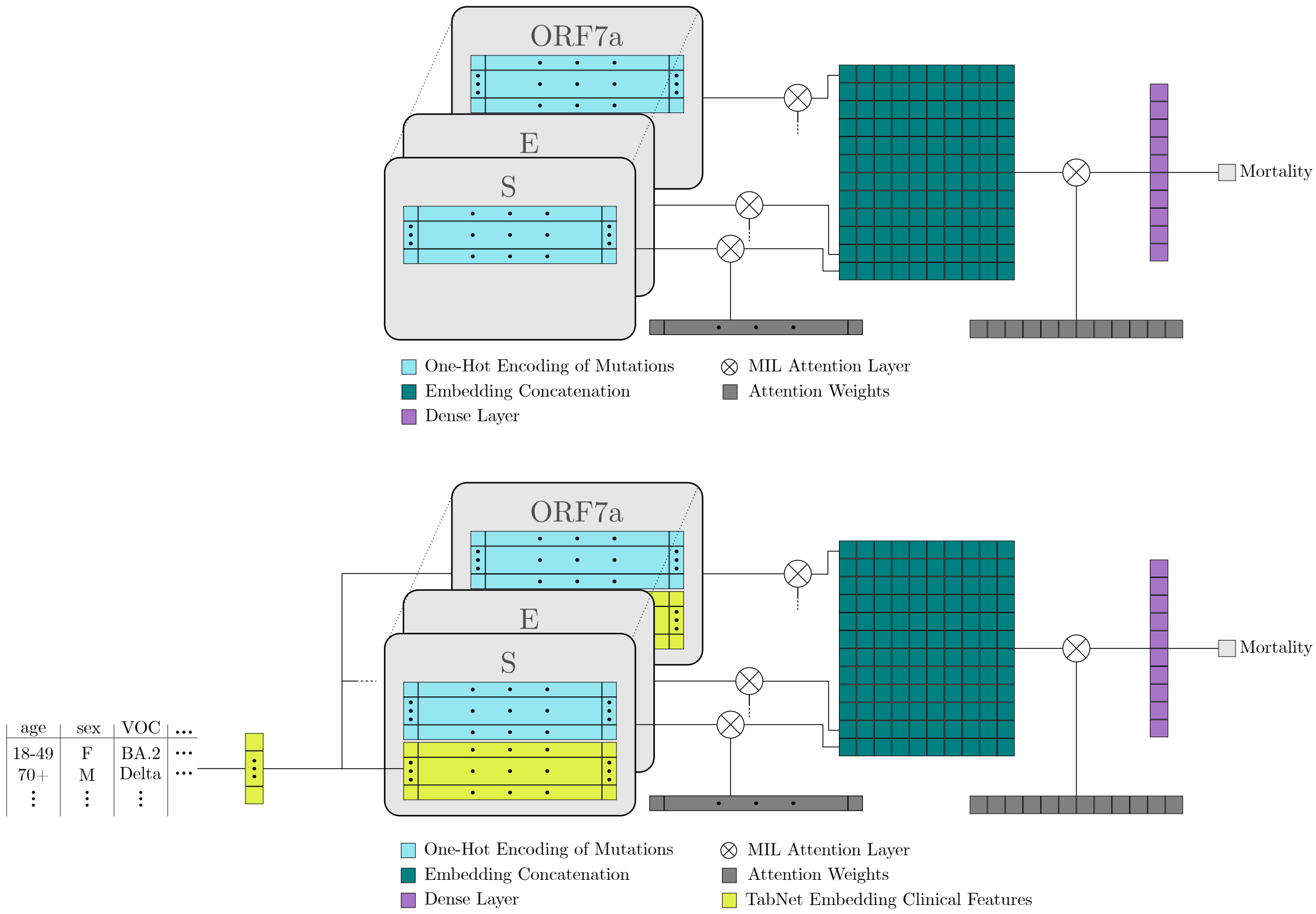

Figure SF6: Visualization of the proposed models. **(A)** is the multilevel model's structure, which learns directly from mutation positions. We create an embedding matrix for each protein by encoding every mutation position as a one-hot encoded vector. These embeddings are forwarded through a MIL attention layer to produce local attention weights for each mutation in each protein. The second MIL attention layer produces interpretations across the embeddings from the previous attention layers, indicating which protein is more relevant for the final severity prediction. **(B)** ClinIAN extends this structure by adding a TabNet in front of the model; this TabNet produces an embedding of the clinical features and interpretations of the clinical input features. For each mutation position, this embedding is added, enabling the model to take this information into account for each position.

We present the full structure of both the Mutation Model and ClinIAN in Figure SF6. We further display the calculation of the protein embedding $\tilde{z}$ and the protein embeddings a_p in the equations SE1, SE2.

$$\tilde{z} = \sum_{p \in \{S, \ldots, ORF7a\}} a_p z_p \qquad \text{(SE1)}$$

and with the added gating approach

$$a_p = \frac{\exp\{w^T(\tanh(Vz_p^T) \odot sigm(Uz_p^T))\}}{\sum_{j \in \{S,\dots,ORF7a\}} \exp\{w^T(\tanh(Vz_p^T) \odot sigm(Uz_p^T))\}} \tag{SE2}$$

S2.2 Combining Sequence Data with Clinical Information

We optimize ClinIAN using the suggested sparsity regularization by Arik and Pfister [2021].

With

$$L = L_{\text{prediction}} - \lambda L_{\text{sparsity}} \tag{SE3}$$

and

$$L_{\text{sparsity}} = \sum_{i=1}^{N_{\text{steps}}} \sum_{b=1}^{B} \sum_{j=1}^{D} \frac{-\mathbb{M}_{b,j}[i] \log(\mathbb{M}_{b,j}[i] + \epsilon)}{N_{\text{steps}} \cdot B} \tag{SE4}$$

Where ϵ is introduced for numerical stability, N_{steps} specifies the depth of TabNet, B is the batch size, D is the feature dimensionality, and $M[i] \in \mathbb{R}^{B \times D}$ is a learnable mask for sparse selection of most salient features at step i.

S3 Predicting COVID-19 mortality

All models shown in the paper were evaluated using 10x10 Nested Cross-Validation with a final refit on the entire dataset according to matthews correlation coefficient (MCC). In the following section, we specify the grid parameters used in the evaluations. For all baseline models, we consider the class imbalance through setting the parameter *class_weight* to 'balanced'. For the training of ClinIAN, we apply the identified best parameters from the Mutation Model and only grid search over ClinIAN-specific parameters.
Random Forrest (RF)

- *n_estimators*: 1, 100, 500, 1000
- *max_depth*: None, 5, 10
- *max_features*: sqrt, log2

Linear SVM (LinSVM):

- *C*: 0.0001, 0.001, 0.01, 0.1, 1, 10
- *kernel*: linear

Polynomial SVM (PolySVM):

- *C*: 0.0001, 0.001, 0.01, 0.1, 1, 10
- *kernel*: poly
- *degree*: 2, 3, 4, 5

Mutation-based Model (MutModel):

- ClassBalanceLoss

- *lr*: 0.1, 0.01, 0.001, 0.0001
- *criterion__loss_type*: cross_entropy
- *citerion__gamma*: 1
- *citerion__beta*: 0.99999, 0.9999, 0.999, 0.99
- *optimizer__weight_decay*: $1e-6$, $1e-5$, $1e-4$
- *module__attention_params*: [64,1], [128,1], [256, 1], [512, 1]

- Cross Entropy Loss (Class Balancing through Oversampling)
  - *lr*: 0.1, 0.01, 0.001, 0.0001
  - *criterion*: CrossEntropyLoss
  - *optimizer__weight_decay*: $1e-6$, $1e-5$, $1e-4$
  - *module__attention_params*: [64,1], [128,1], [256, 1], [512, 1]
  - *module__shared_representation_size*: 20, 30, 40
  - *iterator_train__sampler*: WeightedRandomSampler, None

ClinIAN:

- ClassBalanceLoss
  - *lr*: 0.1, 0.01, 0.001, 0.0001
  - *criterion__loss_type*: cross_entropy
  - *citerion__gamma*: 1
  - *citerion__beta*: 0.99999, 0.9999, 0.999, 0.99
  - *optimizer__weight_decay*: $1e-6$, $1e-5$, $1e-4$
  - *module__feature_embedding_size*: 8, 16, 32
- Cross Entropy Loss (Class Balancing through Oversampling)
  - *lr*: 0.1, 0.01, 0.001, 0.0001
  - *criterion*: CrossEntropyLoss
  - *optimizer__weight_decay*: $1e-6$, $1e-5$, $1e-4$
  - *module__feature_embedding_size*: 8, 16, 32
  - *iterator_train__sampler*: WeightedRandomSampler, None

$ClinIAN_{prott5}$

- ClassBalanceLoss
  - *lr*: 0.1, 0.01, 0.001, 0.0001
  - *criterion__loss_type*: cross_entropy
  - *citerion__gamma*: 1
  - *citerion__beta*: 0.99999
  - *optimizer__weight_decay*: 0, $1e-2$

- *module__feature_embedding_size*: 512, 1024

- Cross Entropy Loss (Class Balancing through Oversampling)
  - *lr*: 0.1, 0.01, 0.001, 0.0001
  - *criterion*: CrossEntropyLoss
  - *optimizer__weight_decay*: 0, $1e-2$
  - *module__feature_embedding_size*: 512, 1024
  - *iterator_train__sampler*: WeightedRandomSampler, None

MOMA

- *lr*: $5e-2$, $5e-3$, $5e-4$, $5e-5$, $5e-6$, $5e-7$
- *Modules*: 16, 32, 64, 128

Best performing models

RF (Mutations)

- n_estimators: 5
- max_depth: log2
- n_estimators: 1000

RF (Mutations+Metadata)

- n_estimators: 5
- max_depth: sqrt
- n_estimators: 500

LinSVM (Mutations)

- C: 0.0001

LinSVM (Mutations+Metadata)

- C: 0.0001

PolySVM (Mutations)

- C: 0.01
- degree: 2

PolySVM (Mutations+Metadata)

- C: 0.01
- degree: 2

Mutation Model (Mutations)

- ClassBalanceLoss
- lr: 0.01
- criterion_beta: 0.9999
- optimizer__weight_decay: $1e-06$
- module__attention_params: [512,1]

ClinIAN (Mutations+Metadata)

- ClassBalanceLoss
- lr: 0.001
- criterion_beta: 0.9999
- optimizer__weight_decay: $1e-06$
- module__feature_embedding_size: 32

$ClinIAN_{prott5}$ (Mutations+Metadata)

- ClassBalanceLoss
- lr: 0.001
- criterion_beta: 0.99999
- optimizer__weight_decay: $1e-2$
- module__feature_embedding_size: 512

MOMA (Mutations+Metadata)

- lr: $5e-4$
- $Modules$: 16

S3.1 Quantifying classification performances

We show detailed performance information as well as additional performance measures that are not ideal to compare, as they do not account for class imbalances as well as AUPRC and MCC in Table ST1. We calculated p-values using a Wilcoxon signed-rank test by comparing the outer-loop performance of the 10x10 CV for a given model with that of ClinIAN. We further report the 95% confidence intervals (CI).

Table ST1: Comparison of the sequence-based mutation model and ClinIAN against RF (Random Forrest), LinSVM (linear SVM), and PolySVM (polynomial SVM). Performances are reported as the median and standard deviation (SD) across the 10x10 nested CV iterations. We additionally report 95% confidence intervals (CI) and p-values from a Wilcoxon signed-rank test.

	MCC ± SD [CI]; p-value	AUPRC ± SD [CI]; p-value	Accuracy ± SD [CI]; p-value	AUC ± SD [CI]; p-value	F1 ± SD [CI]; p-value	Macro F1 ± SD [CI]; p-value
**Mutations**						
RF	0.14 ± 0.29 [0.07, 0.48]; 0.92	0.13 ± 0.19 [0.11, 0.38]; 0.85	0.83 ± 0.32 [0.44, 0.90]; 0.63	0.60 ± 0.15 [0.54, 0.75]; 0.85	0.23 ± 0.2 [0.14, 0.50]; 1.00	0.49 ± 0.24 [0.34, 0.69]; 0.77
LinSVM	0.14 ± 0.29 [0.07,0.49]; 0.92	0.13 ± 0.19 [0.11, 0.38]; 0.85	0.83 ± 0.33 [0.42, 0.90]; 0.63	0.60 ± 0.15 [0.54, 0.75]; 0.85	0.23 ± 0.26 [0.13, 0.50]; 1.00	0.49 ± 0.25 [0.33, 0.69]; 0.56
PolySVM	0.14 ± 0.29 [0.07, 0.49]; 0.92	0.13 ± 0.19 [0.11, 0.38]; 0.85	0.83 ± 0.33 [0.42, 0.90]; 0.63	0.60 ± 0.15 [0.54, 0.75]; 0.85	0.23 ± 0.26 [0.13, 0.50]; 1.00	0.49 ± 0.25 [0.33, 0.69]; 0.56
$ClinIAN_{seq}$	0.14 ± 0.27 [0.07, 0.46]; 0.0.92	0.12 ± 0.17 [0.11, 0.35]; 0.85	0.83 ± 0.33 [0.43, 0.90]; 0.63	0.60 ± 0.15 [0.54, 0.75]; 0.77	0.23 ± 0.25 [0.14, 0.49]; 1.00	0.49 ± 0.24 [0.33, 0.68]; 0.62
$ClinIAN_{seq;protT5}$	0.05 ± 0.21 [-0.02, 0.29]; 0.32	0.11 ± 0.14 [0.05, 0.26]; 0.43	0.42 ± 0.37 [0.24, 0.77]; 0.12	0.53 ± 0.08 [0.50, 0.62]; 0.06	0.19 ± 0.17 [0.10, 0.35]; 0.38	0.35 ± 0.25 [0.20, 0.55]; 0.08
ClinIAN	–	–	–	–	–	–
MOMA	–	–	–	–	–	–
$ClinIAN_{protT5}$	–	–	–	–	–	–
**Mutations + Metadata**						
RF	0.16 ± 0.27 [0.09, 0.48]; 0.85	0.14 ± 0.18 [0.11, 0.37]; 0.85	0.83 ± 0.31 [0.46, 0.91]; 1.00	0.63 ± 0.13 [0.55, 0.73]; 0.77	0.25 ± 0.25 [0.14, 0.50]; 1.00	0.51 ± 0.24 [0.35, 0.69]; 0.62
LinSVM	0.14 ± 0.23 [0.05, 0.38]; 1.00	0.13 ± 0.14 [0.09, 0.29]; 0.92	0.81 ± 0.32 [0.42, 0.88]; 0.43	0.58 ± 0.15 [0.53, 0.74]; 0.92	0.23 ± 0.22 [0.12, 0.43]; 0.92	0.49 ± 0.23 [0.32, 0.65]; 0.43
PolySVM	0.14 ± 0.22 [0.05, 0.37]; 1.00	0.13 ± 0.13 [0.09, 0.28]; 0.85	0.80 ± 0.32 [0.42, 0.87]; 0.42	0.59 ± 0.14 [0.53, 0.74]; 0.92	0.23 ± 0.21 [0.12, 0.43]; 0.85	0.49 ± 0.22 [0.32, 0.64]; 0.43
$ClinIAN_{seq}$	–	–	–	–	–	–
$ClinIAN_{seq;protT5}$	–	–	–	–	–	–
ClinIAN	0.18 ± 0.13 [0.10, 0.28]; –	0.14 ± 0.06 [0.12, 0.20]; –	0.77 ± 0.19 [0.56, 0.83]; –	0.65 ± 0.09 [0.57, 0.70]; –	0.24 ± 0.12 [0.17, 0.35]; –	0.53 ± 0.11 [0.45, 0.60]; –
MOMA	0.05 ± 0.08 [0.02, 0.14]; 0.05	0.11 ± 0.02 [0.10, 0.13]; 0.08	0.88 ± 0.04 [0.84, 0.89]; <0.05	0.52 ± 0.04 [0.51, 0.56]; <0.05	0.14 ± 0.10 [0.05, 0.20]; <0.05	0.52 ± 0.05 [0.49, 0.56]; 1.00
$ClinIAN_{protT5}$	0.20 ± 0.13 [0.09, 0.27]; 0.77	0.15 ± 0.04 [0.12, 0.18]; 0.85	0.80 ± 0.21 [0.57, 0.87]; 0.43	0.64 ± 0.10 [0.56, 0.69]; 0.38	0.28 ± 0.13 [0.15, 0.34]; 0.62	0.54 ± 0.10 [0.48, 0.62]; 0.65

S3.2 Identifying predictive features

We calculate the distance between expected attentions and observed attentions for mutation positions as follows. For mutation γ we calculate

$$E[a^{\gamma}_{n,p}] = \frac{\sum_{s\in S} w(\gamma, n, p, s)}{|S|} \tag{SE5}$$

with

$$w(\gamma, n, p, s) = \begin{cases} \frac{1}{N_p} & \text{, if } \gamma \in s, \\ 0 & \text{, otherwise} \end{cases} \tag{SE6}$$

$s \in S$. With γ as a specific mutation, $n \in N_p$ the mutation positions in p.

We calculate the expected attentions for proteins $p \in P = \{S, ..., ORF7a\}$:

$$E[a_p] = \frac{1}{|P|} \tag{SE7}$$

For the clinical features we extract the attention maps provided by TabNet and calculate the expected attentions:

$$E[a^{\psi}_{c}] = \frac{\sum_{s\in S} h(\psi, c, s)}{|S|} \tag{SE8}$$

with

$$h(\psi, c, s) = \begin{cases} \frac{1}{|c|} & \text{, if } \psi \in s, \\ 0 & \text{, otherwise} \end{cases} \tag{SE9}$$

ψ is a specific categorical value within category c (e.g., for $c = age$ $\psi \in \{18-49, 50-69, 70+\}$).

Here we provide the interpretation masks from the paper in large

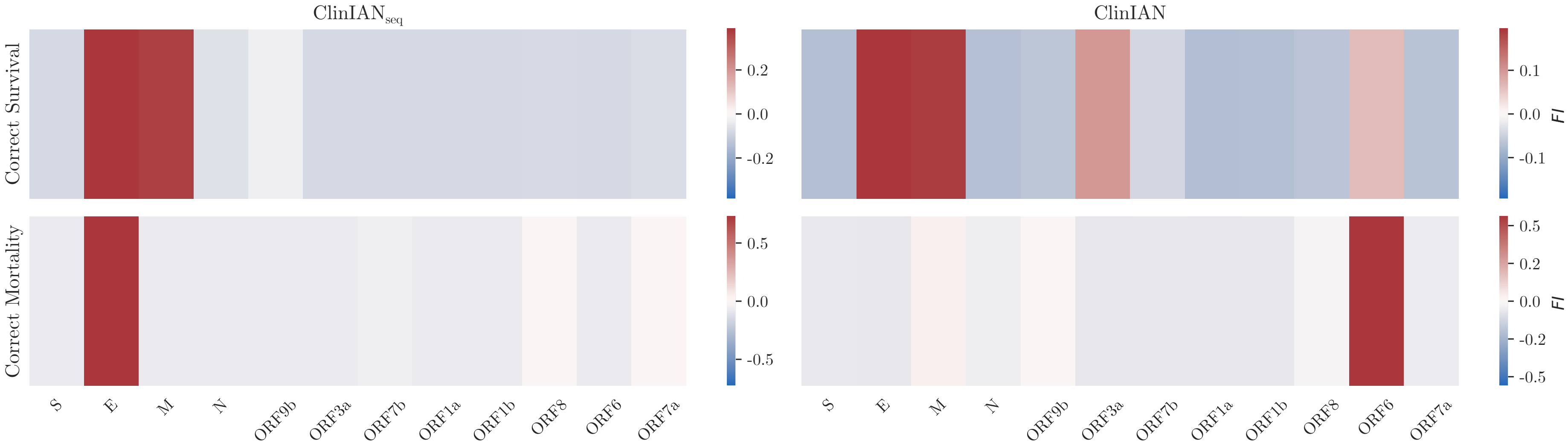

Figure SF7: High Resolution visualization of protein interpretations from the paper.

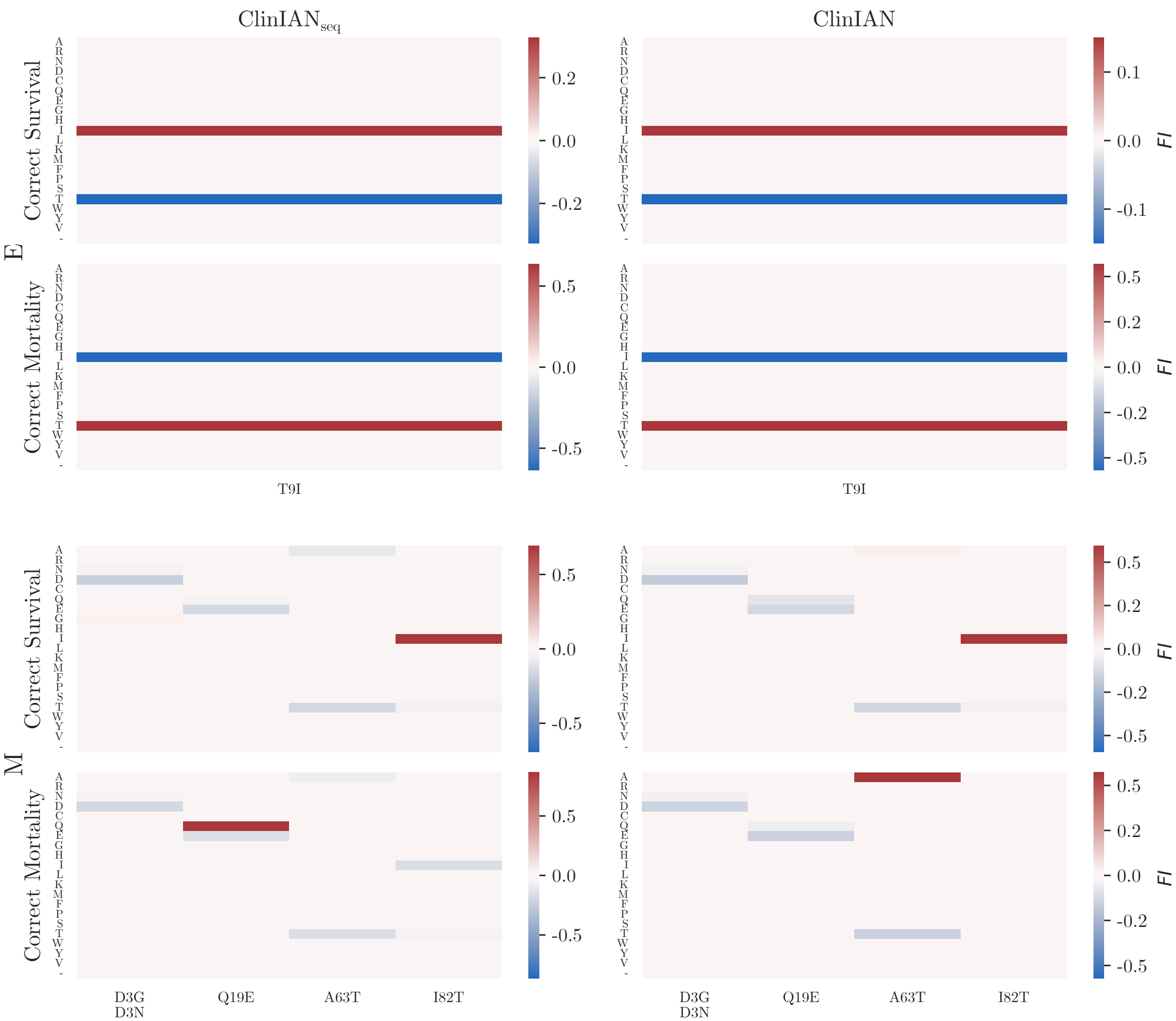

Figure SF8: High Resolution visualization of comparisons from the paper.

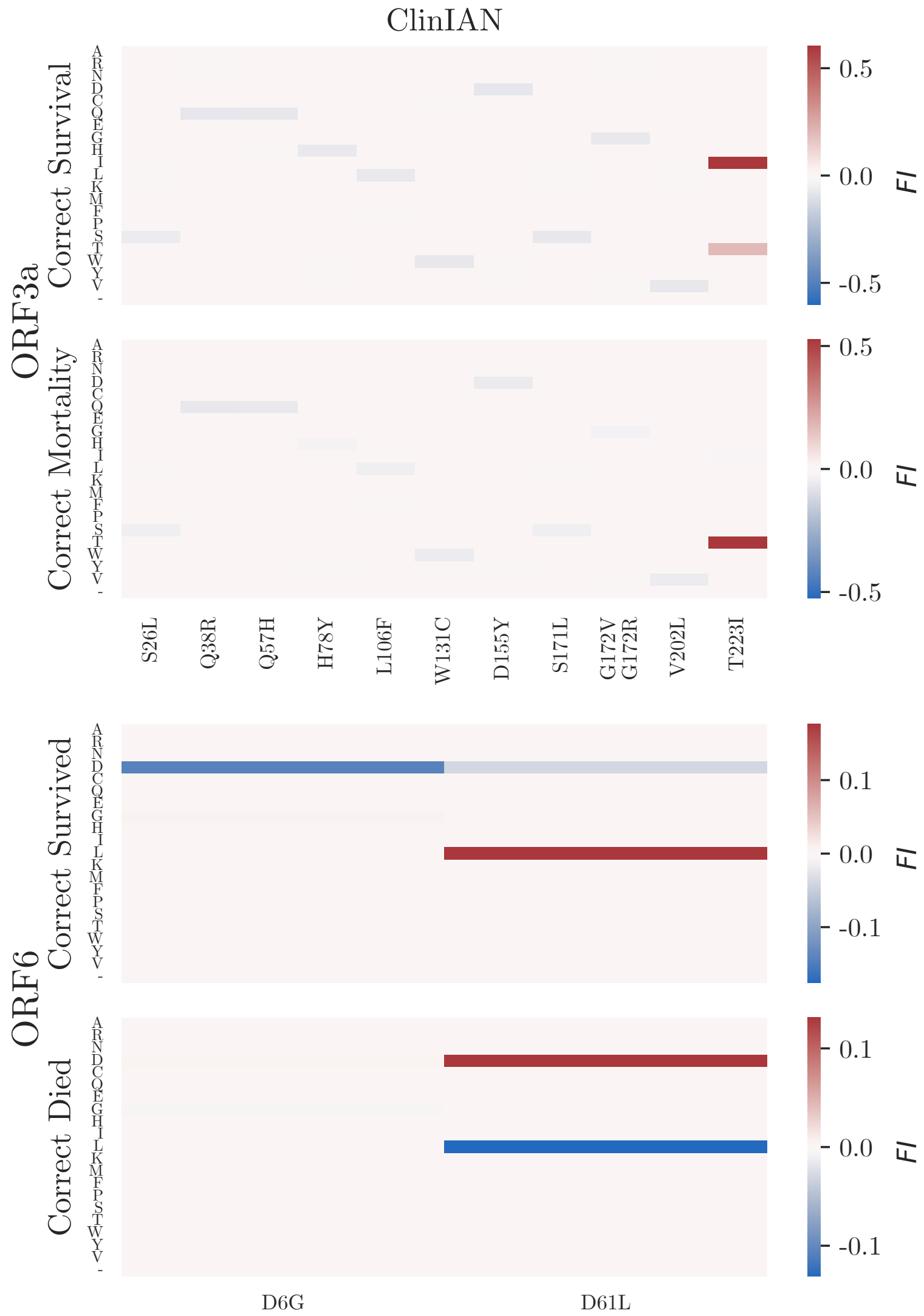

Figure SF9: High Resolution visualization of ORF3a and ORF6 interpretations from the paper.

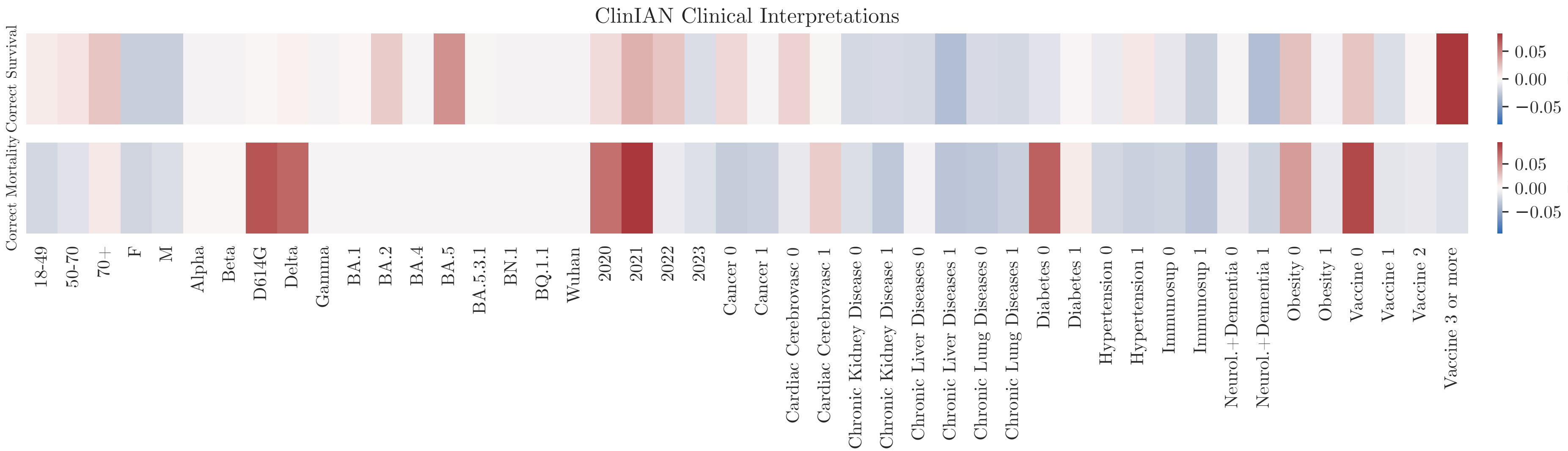

Figure SF10: High Resolution visualization of clinical interpretations from the paper.

S3.3 Directionality Analysis

The FI metric used in our paper helps highlight features that are more predictive than others, but, as with simply using attention weights, it does not indicate whether these features are positively or negatively correlated.

For a directionality analysis, we can decompose the model's predictions using the gradient sign to obtain directionality. This is possible because the projections of our sequence models are linear.

We calculate the positional score $d_{c,p,n}$ as follows:

$$d_{c,p,n} := \alpha_p \cdot \alpha_{n,p} \cdot \mathbf{x}_{p,n}^\top \mathbf{w}_c \quad \text{(SE10)}$$

$$\hat{y}_c = \sum_p \sum_n d_{c,p,n} + b_c \quad \text{(SE11)}$$

With $\hat{y}_c$ being the logit of class c and $p \in P$ being all possible proteins and n being the position within the protein. α_p is the global attention weight and $\alpha_{n,p}$ being the attention for position n in protein p. b_c is the bias term. When we sum over all positions, we get the directionality of the protein p.

$$D_{c,p} := \sum_{n=1}^{N_p} d_{c,p,n} \quad \text{(SE12)}$$

Below, we show the directionality scores for the proteins E, M, ORF6, and ORF3a, as well as the protein-level directionalities.

These results support our assertions in the paper that a positive correlation exists between the identified mutations and proteins and the mortality/survival outcome. Fig. SF11 shows that for outcome mortality, a clear positive correlation exists for protein ORF6. The further decompositions support the paper's assertions about the effects of individual mutations. E.g., a positive correlation between the mutation E:T9I and the outcome survival. Alternatively, a negative correlation between this mutation and mortality.

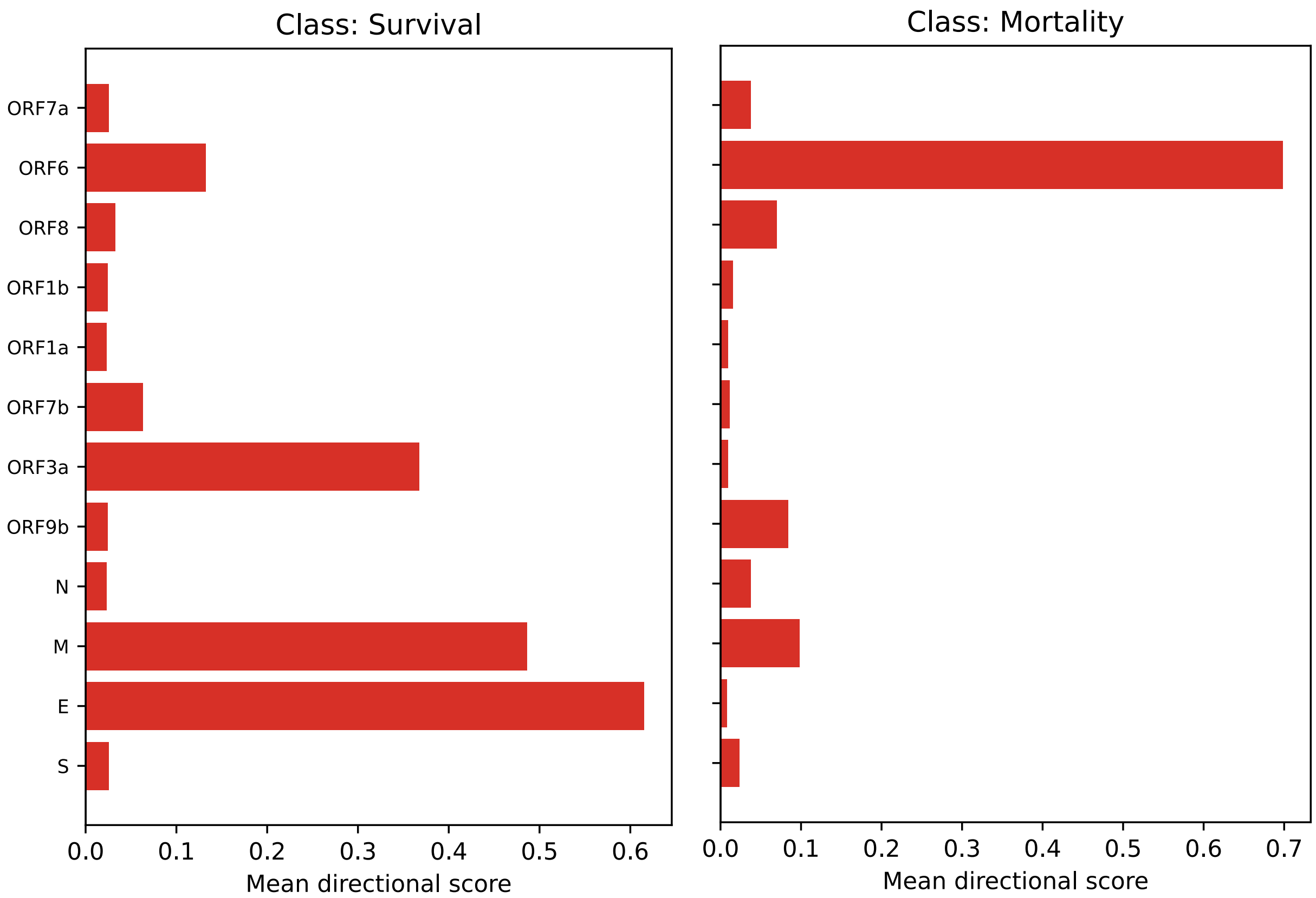

Figure SF11: Directionality score for proteins.

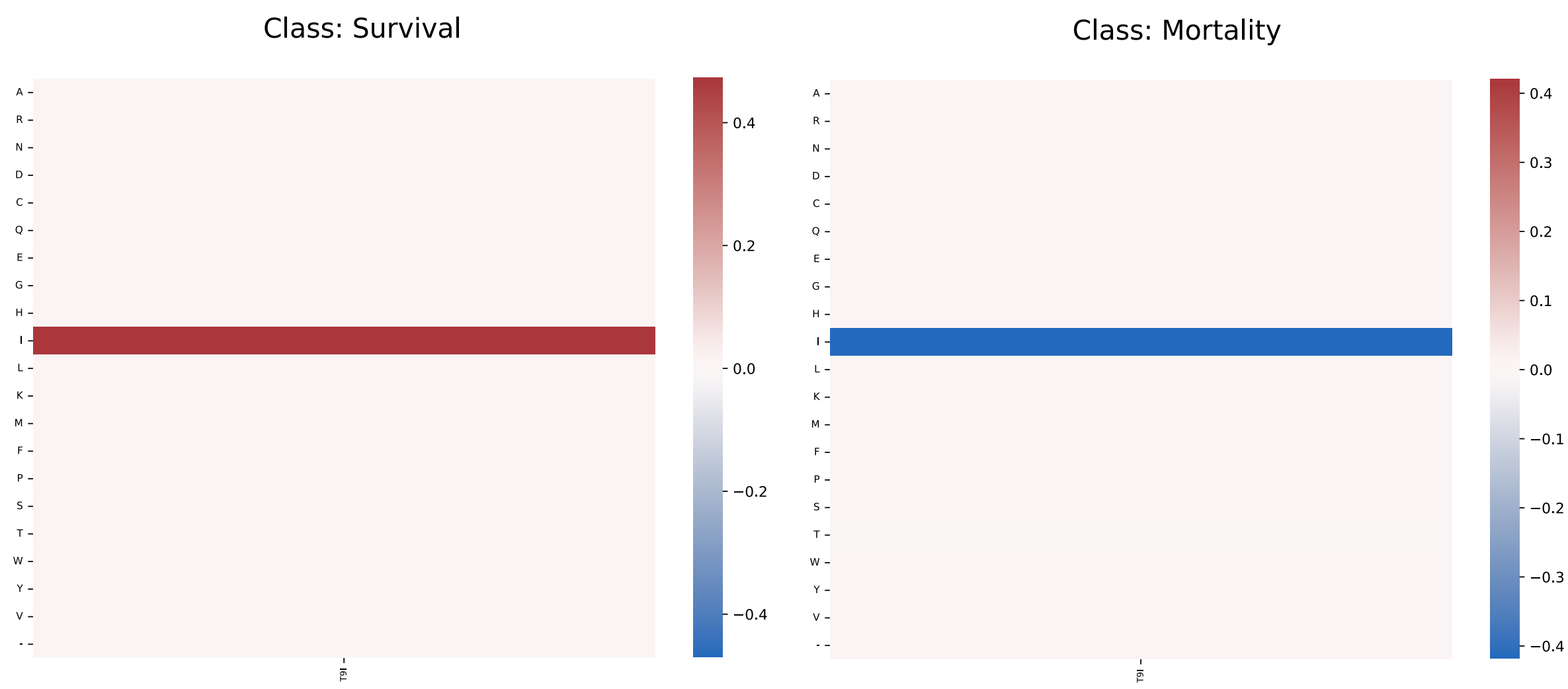

Figure SF12: Directionality score for E protein.

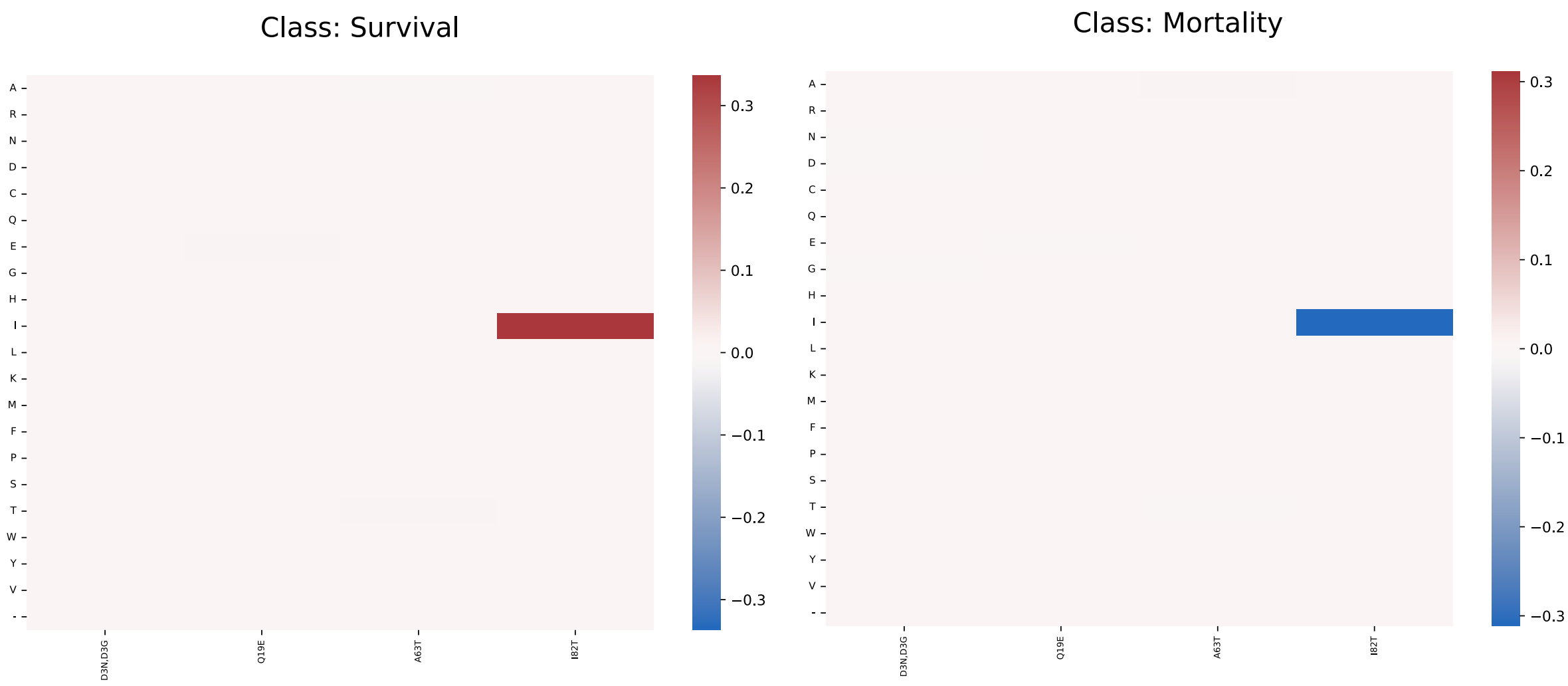

Figure SF13: Directionality score for M protein.

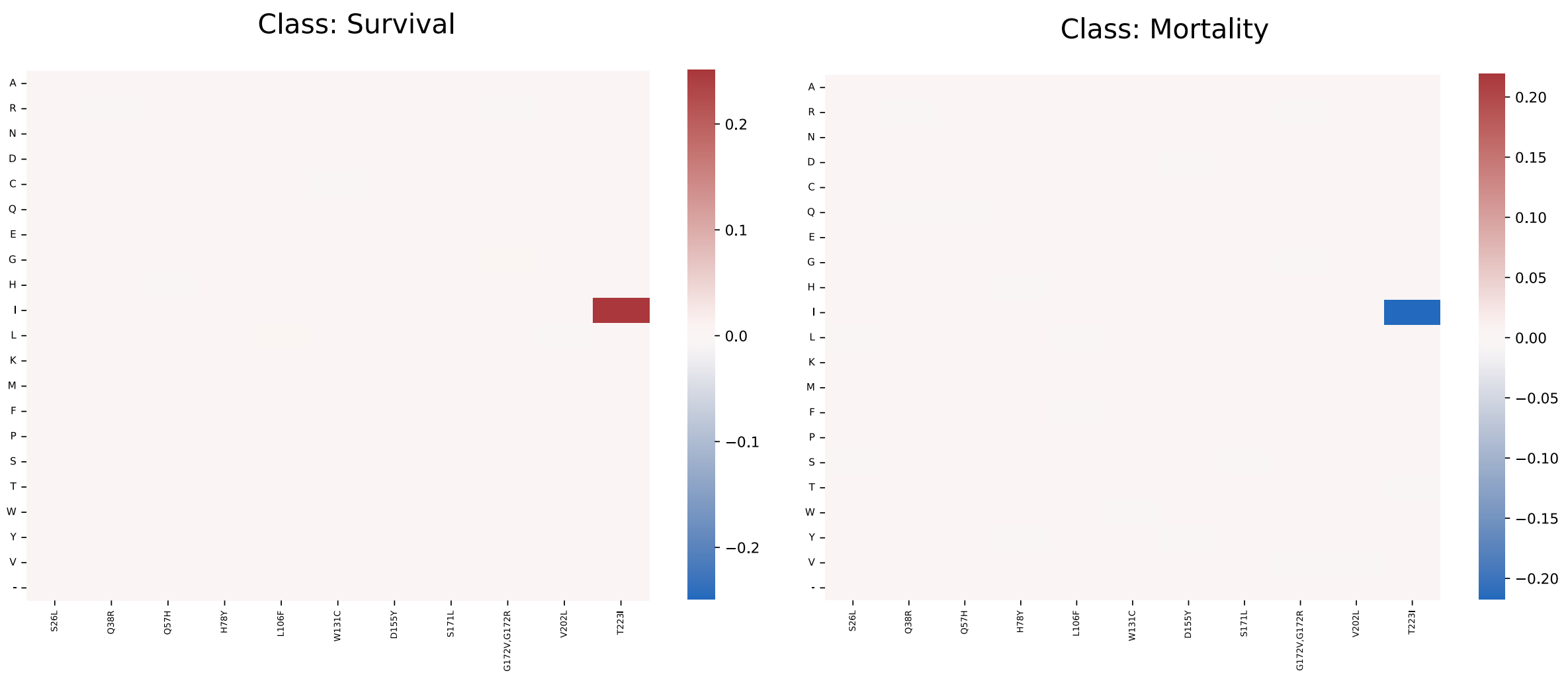

Figure SF14: Directionality score for ORF3a protein.

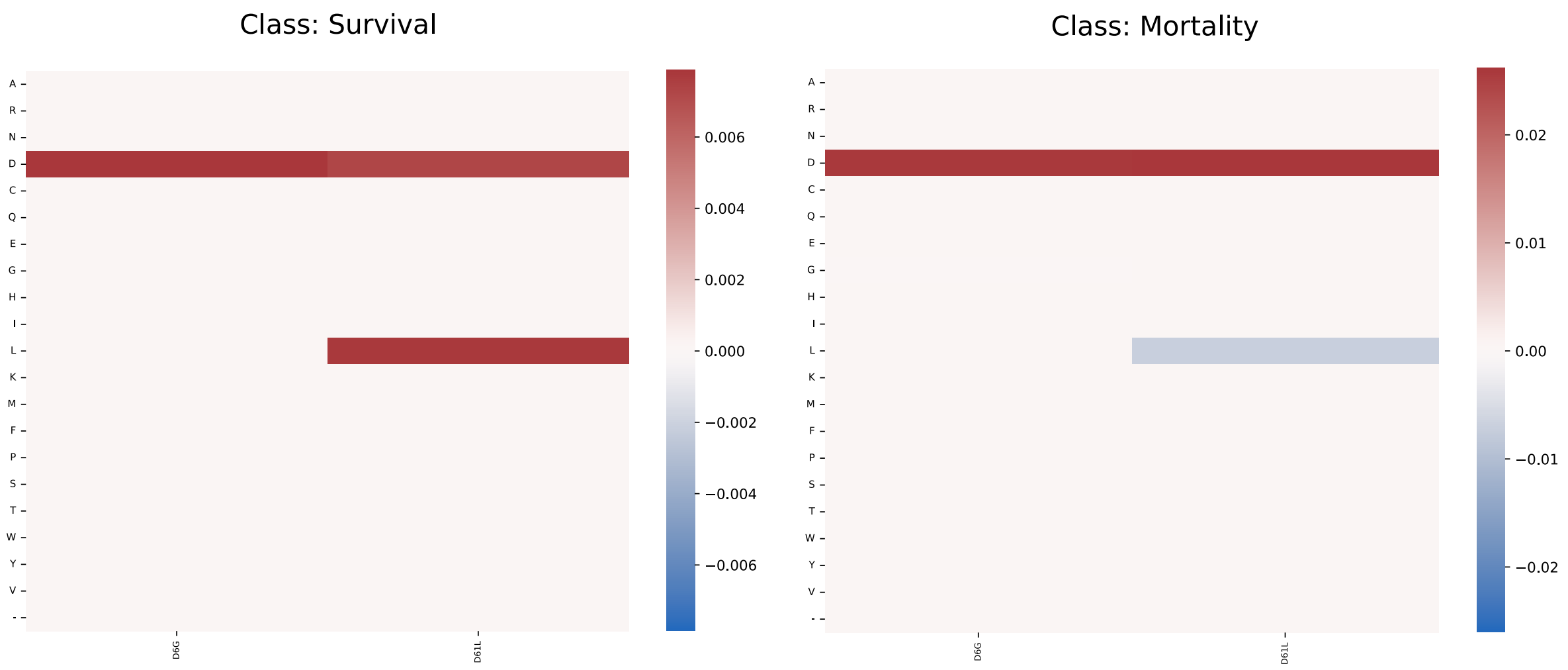

Figure SF15: Directionality score for ORF6 protein.

Table ST2: Ablation study, comparing the performance results of the applied methods in the different settings: Mutations (only sequence data), Metadata (only clinical metadata), Mutations + Metadata (combining clinical metadata with sequence data). We add $ClinIAN_{meta}$, which is just the TabNet performance, into the comparison.

	MCC [CI]	AUPRC [CI]
	**Mutations**	
RF	**0.14** [0.07, 0.48]	**0.13** [0.11, 0.38]
LinSVM	**0.14** [0.07, 0.49]	**0.13** [0.11, 0.38]
PolySVM	**0.14** [0.07, 0.49]	**0.13** [0.11, 0.38]
$ClinIAN_{meta}$	–	–
$ClinIAN_{seq}$	**0.14** [0.07, 0.46]	0.12 [0.11, 0.35]
$ClinIAN_{seq;prott5}$	0.05 [-0.02, 0.29]	0.11 [0.05, 0.26]
ClinIAN	–	–
MOMA	–	–
$ClinIAN_{prott5}$	–	–
	**Metadata**	
RF	**0.28** [0.13, 0.38]	**0.13** [0.11, 0.38]
LinSVM	0.23 [0.12, 0.40]	**0.13** [0.11, 0.38]
PolySVM	0.17 [0.08, 0.30]	**0.13** [0.11, 0.38]
$ClinIAN_{meta}$	0.19 [0.07, 0.30]	0.12 [0.11, 0.35]
$ClinIAN_{seq;prott5}$	–	–
ClinIAN	–	–
MOMA	–	–
$ClinIAN_{prott5}$	–	–
	**Mutations + Metadata**	
RF	0.16 [0.09, 0.48]	0.14 [0.11, 0.37]
LinSVM	0.14 [0.05, 0.38]	0.13 [0.09, 0.29]
PolySVM	0.14 [0.05, 0.37]	0.13 [0.09, 0.28]
$ClinIAN_{meta}$	–	–
$ClinIAN_{seq}$	–	–
$ClinIAN_{seq;prott5}$	–	–
ClinIAN	0.18 [0.10, 0.28]	0.14 [0.12, 0.20]
MOMA	0.05 [0.02, 0.14]	0.11 [0.10, 0.13]
$ClinIAN_{prott5}$	**0.20** [0.09, 0.27]	**0.15** [0.12, 0.18]

Table ST3: Ablation study, comparing the performance results ClinIAN trained on all features and ClinIAN trained on all features except VOC and calendar year.

	MCC [CI]	AUPRC [CI]
ClinIAN	0.18 [0.10, 0.28]	0.14 [0.12, 0.20]
$ClinIAN_{no_VOC_year}$	0.21 [0.10, 0.36]	0.15 [0.10, 0.26]

S3.4 Ablation Study: Clinical vs. Sequence vs. Combined

We perform ablation studies on to identify the performance on the clinical features, the sequence features and the combined features.

From a performance-only perspective, the clinical tabular features perform better. While we can learn a lot from the clinical features, we are interested in building a model that extracts medically relevant information from the sequences while accounting for the effects of the clinical features. We aimed to develop a model that performs at a level comparable to the state of the art while providing a tool for knowledge gain in practical applications.

ClinIAN yields interpretable, clinically adjusted effect estimates for the sequence features, supporting hypothesis generation and mechanistic insight that a clinical-only model cannot provide.

S3.5 Ablation Study: Model Performance without VOC and calendar year

For further evaluation of the influence of VOC and year on the prediction, we train a model on all features except VOC and calendar year.

The results show that, in the absence of the variables VOC and calendar year, we still learn a model with similar performance (slightly better on median performance). This shows that other features are also predictive, but we cannot rule out a high correlation between these variables, since we are no longer correcting for them. In our original approach, we expect that the importance of the other features reflects additional predictive information rather than mere correlation with VOC and calendar year, since we are correcting for these in the analysis.

S3.6 Training Setup: ClinIAN

We train ClinIAN with the defined grid parameters from Sec. S3. Below, we provide the training curve from the 10x10 nested cross-validation setup. As our main focus is maximizing the MCC, which more clearly shows misclassifications of the minority class, we provide a prediction performance curve on the validation split across epochs for all models. We decide on the best model using early stopping on the validation MCC performance, which is the most relevant measure. The spread of performances across all models is indicated in opaque, with the mean performance plotted in greater detail. Due to early stopping, not all models are trained for the same duration, so only one model's performance is shown at the end.

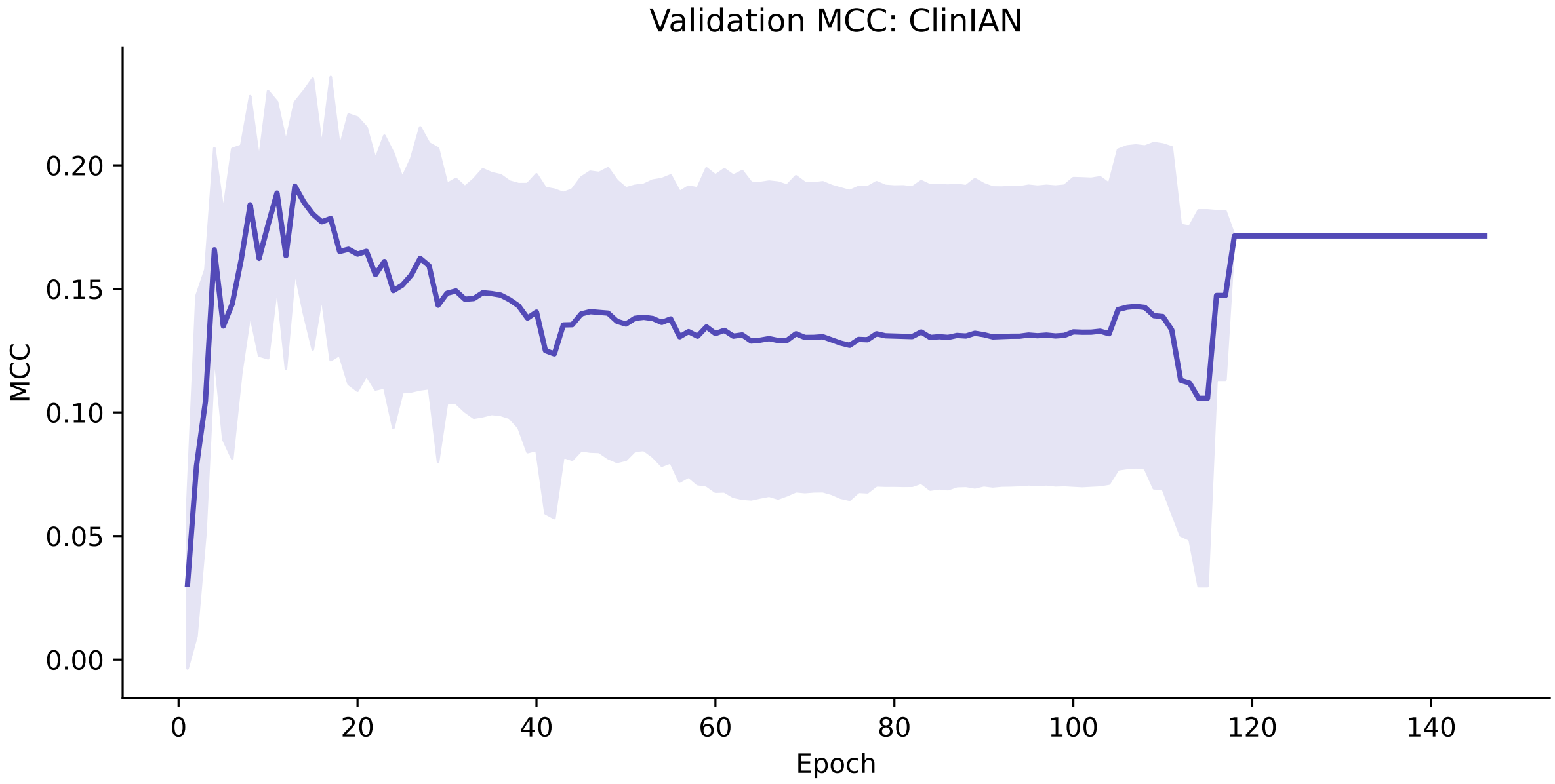

Figure SF16: Line-plot depicting the evolution of the MCC performance on the validation split during the training of ClinIAN. As we use 10x10 nested cross-validation the spread of performances are indicated in opaque, with the mean performance highlighted clearly.

Figure SF16 depicts the training progress for the validation MCC. Initially the validation performance increases nicely, indicating that the model learns from the data. Later the performance drops slightly and plateaus.

S3.7 Statistical Validation

We validated the identified mutations on an external cohort of viral sequences shared via GISAID, the global data science initiative [Khare, 2021]. The used set can be accessed through the EPI_SET ID *EPI_SET_260115ak* or https://doi.org/10.55876/gis8.260115ak. The set contains 117,573 individual viruses from the timeframe 2020-03-01 to 2025-09-25. We preprocessed the not clearly defined patient status by labeling every value that clearly mentioned the patient from whom the sample came as 'passed away', with 'died'. We treated the following patient statuses as the patient passing away:

- *Deceased*
- *deceased*
- *exitus*
- *Dead*
- *Hospitalized (Deceased)*
- *Hospitalized, Deceased*
- *Fatal*
- *Exitus*
- *Diseased*
- *Severe, deceased*
- *Deceased, severe, Horpitalized*
- *Died*
- *DECEASED*
- *Hospitalized (deceased)*
- *Death*

We treated all other samples as if they came from surviving patients. We then applied a Wilcoxon Signed-Rank test to assess the significance of the mutations identified by our model between patients who passed and those who survived. Table ST4 shows the results of this evaluation, with p-values presented with and without correction for multiple testing with Bonferroni correction.

Mutation	p-value	p-value corrected (Bonferroni)
M:Q19E	6.972297085154424e-19	3.486148542577212e-18
M:A63T	8.015086614689961e-19	4.0075433073449806e-18
E:T9I	2.395388427613082e-17	1.197694213806541e-16
ORF3a:T223I	5.176773615595196e-14	2.5883868077975977e-13
ORF6:D61L	5.1350764141959106e-11	2.5675382070979554e-10

Table ST4: Results of the Wilcoxon Signed-Rank test for the mutations described in the manuscript as identified by ClinIAN.

All mutations identified by the model show a significant association with this large external cohort, which validates the interpretative value of our proposed model.

Supplementary Appendix

All genome sequences and associated metadata supporting the findings of this study can be accessed through the persistent digital object identifier https://doi.org/10.55876/gis8.260115ak

In addition to the minted DOI, GISAID also communicates the aggregation of GISAID accession numbers (EPI_ISL_IDs) through the corresponding EPI_SET_260115ak identifier to facilitate both, the acknowledgment of all data contributors and the direct retrieval of the underlying data from GISAID used in this study.

hCoV-19 Virus Data Summary

GISAID Identifier	Digital Object Identifier	Number of individual viruses	Data Collection range	Number of countries/territories
EPI_SET_260115ak	https://doi.org/10.55876/gis8.260115ak	117,573	2020-03-01 to 2025-09-25	45

S3.8 MOMA - A Multi-task Attention Learning Algorithm for Multi-omics Data Interpretation and Classification

To compare against established methods, we include the state-of-the-art method MOMA [Moon and Lee, 2022]. We implement MOMA as shown in the github[1]. To ensure fairness in the evaluation, we evaluate the model exactly as described in the GitHub page and ensure the same datasplits for the nested 10x10 CV. As described in their example we train MOMA with BCELoss. We describe the grid parameters and the final optimal parameters in Sec S3. We train the model as described with 500,000 epochs and early stopping. To be consistent with all other models we define early stopping not on the loss as described in their example implementation but on the validation MCC. We also incorporate a class weight to adjust for the class imbalances.

S4 Reproducibility

To reproduce the analysis results, the code is available on GitHub: pfeiferAI/ClinIAN with the specific commit hash fd9ba9715c4189b462b727ed2533d5fbbe211c5e.

[1] `https://github.com/dmcb-gist/MOMA`

References

S. Ö. Arik and T. Pfister. Tabnet: Attentive interpretable tabular learning. In *Proceedings of the AAAI conference on artificial intelligence*, volume 35, pages 6679–6687, 2021. doi: 10.1609/aaai.v35i8.16826.

e. a. Khare. Gisaid's role in pandemic response. *China CDC weekly*, 3(49):1049, 2021. doi: 10.46234/ccdcw2021.255.

S. Moon and H. Lee. Moma: a multi-task attention learning algorithm for multi-omics data interpretation and classification. *Bioinformatics*, 38(8):2287–2296, 2022. doi: 10.1093/bioinformatics/btac080.
